# Phosphoproteomic analysis reveals Smad protein family activation following Rift Valley fever virus infection

**DOI:** 10.1371/journal.pone.0191983

**Published:** 2018-02-06

**Authors:** Cynthia de la Fuente, Chelsea Pinkham, Deemah Dabbagh, Brett Beitzel, Aura Garrison, Gustavo Palacios, Kimberley Alex Hodge, Emanuel F. Petricoin, Connie Schmaljohn, Catherine E. Campbell, Aarthi Narayanan, Kylene Kehn-Hall

**Affiliations:** 1 National Center for Biodefense and Infectious Diseases, School of Systems Biology, George Mason University, Manassas, Virginia, United States of America; 2 United States Army Medical Research Institute of Infectious Diseases, Frederick, Maryland, United States of America; 3 Center for Applied Proteomics and Molecular Medicine, School of Systems Biology, George Mason University, Manassas, Virginia, United States of America; 4 DCE Consulting, Vienna, Virginia, United States of America; School of Veterinary Medicine University of California Davis, UNITED STATES

## Abstract

Rift Valley fever virus (RVFV) infects both ruminants and humans leading to a wide variance of pathologies dependent on host background and age. Utilizing a targeted reverse phase protein array (RPPA) to define changes in signaling cascades after *in vitro* infection of human cells with virulent and attenuated RVFV strains, we observed high phosphorylation of Smad transcription factors. This evolutionarily conserved family is phosphorylated by and transduces the activation of TGF-β superfamily receptors. Moreover, we observed that phosphorylation of Smad proteins required active RVFV replication and loss of NSs impaired this activation, further corroborating the RPPA results. Gene promoter analysis of transcripts altered after RVFV infection identified 913 genes that contained a Smad-response element. Functional annotation of these potential Smad-regulated genes clustered in axonal guidance, hepatic fibrosis and cell signaling pathways involved in cellular adhesion/migration, calcium influx, and cytoskeletal reorganization. Furthermore, chromatin immunoprecipitation confirmed the presence of a Smad complex on the interleukin 1 receptor type 2 (IL1R2) promoter, which acts as a decoy receptor for IL-1 activation.

## Introduction

The four basic tenets of viral pathogenesis are the ability to enter host cells, cause productive replication, interfere with host innate or adaptive immune responses, and damage the host [1]. Although the main Rift Valley fever virus (RVFV) determinants of virulence have been mapped to its nonstructural protein (NSs) [2–5] and its antagonism of the interferon response, the full spectrum of host factors involved, especially in regards to host damage, is not well defined. Identification of host alterations during viral infection using global omics-based technologies have increased our understanding of the host-pathogen interface, in addition to yielding diagnostic biomarkers and new therapeutic targets (as reviewed in [6]). Consequently, we performed an analysis of attenuated and virulent RVFV strains utilizing reverse phase protein array (RPPA) to identify phospho-signaling changes associated with early, mid and late phases of infection.

RVFV, a member of the *Phlebovirus* genera, is a tri-segmented RNA virus that causes severe morbidity and mortality in livestock animals [7–11]. RVFV infection in humans leads to clinical symptoms ranging from mild febrile illness to hepatitis, retinitis, delayed-onset of encephalitis, or in more severe cases, hemorrhagic fever and death. Transmission occurs by mosquito bite or contact with infected animal fluids or tissues [12]. Outbreaks have thus far been limited to the African continent and Arabian Peninsula. However, the presence of domestic mosquito species capable of replicating RVFV and lack of prophylactic therapies or licensed vaccines for human use has raised concerns over introduction into the United States [12–14].

Although a comparison of the temporal-dependent phosphorylation profile of 113 host proteins by RPPA did not reveal differences between the attenuated and virulent strains of RVFV, a high induction of phosphorylation of Smad proteins during RVFV infection was observed. The Smad proteins are an evolutionarily conserved transcription factor family with homologs in *Drosophila* (the protein mothers against decapentaplegic (MAD)) and *Caenorhabditis elegans* (the protein SMA (from gene *sma* for small body size)), reviewed in [15–19]. They are primarily phosphorylated by and transducers of transforming growth factor-beta (TGF-β) or bone morphogenetic protein (BMP) receptor mediated signaling. So far, eight Smad family members had been characterized with distinct functions. Receptor regulated (R)-Smads (Smad1, -2, -3, -5, and -8/9) contain two globular domains, MH1 and MH2, connected by a linker region. Smad1/5/9 and Smad2/3 are primarily dependent on BMP and TGF-β type I/II receptor activation, respectively, although TGF-β can stimulate Smad1/5/9 as well. The R-Smad MH2 domain contains a Ser-Ser-X-Ser motif in which the second and third serine residues are phosphorylated after receptor activation. Negative regulator Smads (I-Smad6 and -7) interfere with Smad-receptor or Smad-Smad interactions and lack an MH1 domain. Smad4, also known as Co-Smad, interacts with all R-Smads forming a heterotrimer and mediates nucleocytoplasmic shuttling.

From our study, R-Smads (Smad1/5/9 and Smad2) demonstrated more than 2-fold increase in phosphorylation that was dependent on active RVFV replication. Knockdown of R-Smads alone or in combination with Smad4 had no direct effect on RVFV replication. Potential Smad-regulated promoters were identified through promoter analysis of differentially expressed cellular genes after RVFV infection. Interleukin 1 (IL1) decoy receptor, IL1R2, was observed to be highly expressed and contained increased promoter associated Smad4 as determined by chromatin immunoprecipitation (ChIP). The mechanism and impact of R-Smad activation on RVFV pathogenesis are discussed within.

## Materials and methods

### Cell culture

Vero (ATCC, Manassas, VA) were maintained in DMEM containing 1% L-glutamine and 10% FBS. The human small airway epithelial cells (HSAECs) were grown in Ham’s F12 media supplemented with 1% L-glutamine, 1% penicillin/streptomycin, 1% nonessential amino acids, 1% sodium pyruvate, 10% FBS, and 0.1% 55mM β-mercaptoethanol (Life Technologies; [20]). The human hepatoma cell line Huh-7 was maintained in DMEM containing 1% L-glutamine, 1% nonessential amino acids, 1% sodium pyruvate, 10% FBS. All cells were maintained at 37°C in a humidified 5% CO_2_ atmosphere.

### RVFV viral stocks

Recombinant RVFV viruses were rescued by transfection as previously described [21–25]. A passaged (P1) viral stock was generated by infecting subconfluent Vero monolayers at a multiplicity of infection (MOI) 0.1. Two days post infection media supernatants were harvested several times, to increase the volume and yield of virus harvested, and stored at 4°C. After the last collection, supernatants were pooled together, 0.22μM filtered, and stored at -80°C in aliquots. Infectious viral titers were determined by plaque assay on Vero cells. All work involving select agents is registered with the Centers for Disease Control and Prevention and conducted at George Mason University’s Biomedical Research Laboratory, which is registered in accordance with Federal Select Agent regulations.

### UV inactivation

To generate UV inactivated MP12 and ZH548 viruses, viral supernatants were exposed to five energy doses of 120,000 μJoules each. Supernatants were mixed after every second dose to ensure even inactivation. Supernatants were then pooled, aliquoted and stored at -80°C. Infectious viral titers were determined by plaque assay on Vero cells with a dilution range of undiluted to 10^−3^. Virus stocks were considered inactive if there were no plaques in the undiluted sample.

### Viral infections and sample harvest

HSAEC cells (7.5 x 10^5^ cells/well) were seeded into 6-well plates. Infections with MP12 and ZH548 viruses were performed at an MOI 5. An equal quantity of UV inactivated viral supernatants was used to generate control lysates. After one hour incubation, virus inoculum was removed, cells were washed three times with phosphate buffered saline without Ca^2+^ and Mg^2+^ (PBS; Gibco-Invitrogen) and cultured until harvest. Conditioned, filtered media was used for all infections and subsequent culturing to limit potential activation of signaling cascades due to fresh serum. Mock samples were treated similarly with conditioned media alone. The same conditions for infection and harvest were used for Huh-7 produced samples.

Samples were harvested at 1, 3, 9, and 18hpi. Extracellular supernatants were clarified by centrifugation, aliquoted, and stored at -80°C. Cells were then washed once with PBS and either lysed directly in blue lysis buffer, TRIzol LS (ThermoFisher Scientific) or trypsinized for flow cytometry analysis. Blue lysis buffer (1.25:1 ratio of 2x Novex^®^ Tris-Glycine SDS Sample Buffer and Tissue Protein Extraction Reagent (T-PER; ThermoFisher Scientific), 2.1mM EDTA pH 8.0, 0.85mM NaF, 170μM Na_3_VO_4_, 27.7mM dithiothreitol (DTT), and EDTA-free Complete Protease Inhibitor Cocktail tablet (Roche)) or TRIzol LS was added to cells directly in wells for five minutes with occasional rocking. Samples were then scrapped and transferred to Sarstedt tubes. Blue lysis samples were incubated at 95°C for 15 mins to inactivate virus. Both TRIzol LS and blue lysis samples were stored at -80°C until further processing or analysis.

### Viral kinetics

Extracellular media supernatants were collected at various times post-infection and infectious viral titers were determined by plaque assay on Vero cells [26].

Intracellular RNA was extracted from Trizol LS lysates using the Direct-zol RNA MiniPrep kit (Zymo Research) with an on-column DNase I digestion step and eluted in 1mM sodium citrate (RNA Storage Solution, ThermoFisher Scientific). Absolute quantification of RVFV genomic copies was determined by RT-quantitative (q)PCR utilizing the TaqMan^®^ RNA-to-CT^™^ 1-Step Kit (ThermoFisher Scientific). Fifty nanograms of total RNA was utilized per reaction. Twenty microliter reactions were setup according to manufacturer’s recommendations with 0.2μM RVFV probe (6FAM-AAAGCTTTGATATCTCTCAGTGCCCCAA-TAMRA) with 0.2μM M segment primers (Forward 5’-AAAGGAACAATGGACTCTGGTCA-3’ and Reverse 5’- CACTTCTTACTACCATGTCCTCCAAT-3’). Reactions conditions were as follows: hold at 48°C for 15min, hold at 95°C for 10min, and 40 cycles at 95°C for 15sec and 60°C for 1min. Viral genomic copies were determined by comparing the unknowns to a standard curve containing known RNA quantities and extrapolating the value. RNA standards were made by extracting viral RNA from RVFV MP12 viral supernatants using TRIzol LS Reagent and quantifying the viral RNA with Quant-iT Ribogreen RNA assay kit (Thermo-Fisher Scientific).

For flow cytometric analysis, HSAECs were trypsinized at various time points after infection and fixed in 4% paraformaldehyde for 15min on ice. Next, cells were washed twice with chilled PBS and stored in FACs buffer (PBS with 1% FBS) at 4°C until all samples were collected. Cells were permeabilized in PBS with 0.2% Triton X100 for 15min on ice and then incubated overnight with RVFV nucleoprotein (NP) antibody (clone 1D8 [1:1000]; BEI Resources) in FACs buffer. Cells were then incubated for 1–2 hours at room temperature with secondary antibody donkey anti-mouse IgG conjugated to Alexa Fluor 488 (1:1000; ThermoFisher Scientific). Percentage of RVFV NP positive cells from 10,000 cells was determined using EMD Millipore Guava^®^ easyCyte HT Sampling Flow Cytometer.

### Reverse phase protein array (RPPA)

Cell lysates were immobilized onto nitrocellulose coated slides (Grace Bio-Labs) using an Aushon 2470 arrayer (Aushon BioSystems). Each sample was printed in triplicate along with standard curves for internal quality control. Selected arrays were stained with Sypro Ruby Protein Blot Stain (Life Technologies) following manufacturing instructions in order to quantify the amount of protein present in each sample [27]. The remaining arrays were treated for 15 minutes at room temperature with the mild stripping reagent, Reblot Antibody Stripping solution (EMD Millipore), in order to expose antigenic sites prior to antibody staining. The arrays were then washed twice for 5min at room temperature in PBS) (Life Technologies), and incubated for 5h in I-Block (Applied Biosystems) in order to block non-specific binding sites on the nitrocellulose. Using a Dako Autostainer Universal Staining System, arrays are first probed with 3% hydrogen peroxide, biotin blocking system (Dako Cytomation), and an additional serum free protein block (Dako Cytomation) to reduce non-specific binding between endogenous proteins and the detection system.

Arrays were probed with 113 antibodies, of which 90 were phospho-specific. The majority of the antibodies were purchased from Cell Signaling Technologies (CST), while the following were purchased from other vendors: Beclin 1 (#PRS3613, Sigma), Histone H3 S10 and S28 (#06–570 and 07–145, Upstate Biotechnology), IL10 (#ab52909, Abcam), IL6 (#5143–100, BioVision), IL8 (#ab7747, Abcam), MMP-11 (#ab52904, Abcam), p27 T187 (#71–7700, Zymed), PDGFR Y716 (#07–021, Upstate Biotechnology), PKC alpha S657 (#06–822, Upstate Biotechnology), Protein Phosphatase 1 beta (#ab53315, Abcam), Ron Y1353 (#5176–1, Epitomics), and S100A7 Calcium BP (#H00006278-A01, Abnova). Antibodies were validated for their use on the array by Western Blot to determine antibody specificity. Only antibodies showing a single band at the expected molecular weight were used on the arrays [28]. Biotinylated anti-rabbit (Vector Laboratories, Inc.) or anti-mouse secondary antibody (CSA; Dako Cytomation) was used in conjunction with GenPoint^™^ kit (Dako Cytomation), a commercially available tyramide-based signal amplification system. Fluorescent detection was obtained through the use of IRDye 680RD Streptavidin (LI-COR Biosciences) according to the manufacturer’s recommendations. Antibody and Sypro Ruby stained slides were scanned on a Tecan laser scanner (TECAN, Mönnedorf, Switzerland) using the 620nm and 580nm weight length channel, respectively.

Images were analyzed with MicroVigene Software Version 5.1.0.0 (Vigenetech). The software performs spot finding along with subtraction of the local background and non-specific binding generated by the secondary antibody. Each sample signal was normalized to the corresponding amount of protein derived from the Sypro Ruby stained slides and triplicates were averaged. All RPPA data was annotated with Uniprot IDs and total protein or phospho-protein was noted. The average fold change and t-test was calculated for the matched triplicate samples for each RVFV-infected/mock comparison per time point. A t-test was also performed on the triplicate samples for each infected/mock set per time point.

### Protein lysates and western blot

For cellular fractionation, cells were harvested at the indicated time points and processed as detailed before [24]. 10ug of cytoplasmic or nuclear extracts were used for western blot analysis. Blue lysis samples were thawed and boiled for 5min. Lysates were then loaded on a 4–12% Bis-Tris Protein mini gel (1.0 mm; Life Technologies) and transferred to a polyvinylidene difluoride (PVDF) membrane, as previously described [24]. After blocking in 3% skim milk–PBS with 0.1% Tween-20, blots were incubated overnight at 4°C with primary antibodies against RVFV nucleoprotein (clone 1D8 [1:500]; BEI Resources), total Smad 2 (# 5339 [1:1000]; CST), total Smad1 (#9743 [1:1000]; CST), total Smad5 (#12534 [1:500]; CST), phospho-Smad1/5 (S463/465)/9 (S465/467) (#13820 [1:500]; CST), phospho-Smad1/5 (S463/465) (#9516 [1:1000]; CST), phospho-Smad2 (S245/250/255) (#3104 [1:500]; CST), phospho-Smad2 (S465/467) (#3108 [1:500]; CST), lamin A/C (2032 [1:1000]; CST), GAPDH (D16H11 XP [1:1000]; CST) or β-actin-HRP conjugated (AC-15 [1:10,000]; Abcam). The next day, blots were incubated with appropriate secondary antibodies, goat α-mouse or goat α-rabbit IgG (ThermoFisher Scientific 1:2,000). Blots were developed using SuperSignal West Femto chemiluminescent substrate kit (ThermoFisher Scientific) on a Bio-Rad Molecular Imager ChemiDoc XRS system.

### Transfections

For NSs expression studies, Vero cells seeded in 6-well plates (5x10^5^ cells/well) were transfected with media alone, 2.5 μg pcDNA3.1 or 2.5 μg pI.18-RVFV-NSs-Flag pcDNA-NSs-Flag using Attractene transfection reagent (Qiagen). At 24 h post transfection (hpt), cells were lysed in blue lysis buffer, as described above, and analyzed by western blot for protein expression. For siRNA experiments, HSAECs seeded in 6-well plates (3x10^5^ cells/well) were transfected with media alone (Mock) or siRNAs against non-targeting control (NTC; Dharmacon #D-001810-01-05), Smad1 (L-012723-00-0005), Smad2 (L-003561-00-0005) or Smad4 (L-003902-00-0005). All siRNA transfections were performed with Dharmafect Reagent 1 and 50nM of each siRNA either alone or in combination. After 24 hpt, transfection media was removed and fresh complete media was added. At 48 hpt, cells were then infected with MP12 at an MOI 0.1. Extracellular media supernatants and protein lysates were collected and analyzed for viral titers and protein expression, respectively.

### Quantitative (q)PCR analysis

Complementary (c)DNA was generated from RNA using the High Capacity RNA to cDNA kit (ThermoFisher Scientific). Relative gene expression changes were assessed by TaqMan Gene Expression assay (ThermoFisher Scientific) with probe/primers for Smad1 (Hs01077084_m1), Smad2 (Hs00183425_m1), Smad3 (Hs00969210_m1), Smad4 (Hs00929647_m1), Smad5 (Hs00195437_m1), Smad6 (Hs00178579_m1), Smad7 (Hs00998193_m1), IL1R2 (Hs00174759_m1), IL1RL1 (Hs00249384_m1), VAV3 (Hs00916818_m1) and 18S rRNA (Hs99999901_s1). All assays were performed using the ABI StepOne Plus instrument. Fold changes for all conditions were first determined relative to 18S rRNA values and then normalized to mock at each timepoint using ΔΔCt method.

### Promoter and Ingenuity Pathway Analysis

To identify possible Smad-dependent transcripts, RVFV and mock infected normalized RNASeq datasets (GEO Submission # GSE102481 [29]) were utilized for promoter analysis. Average fold changes (RVFV versus mock infected) for each time point from three independent experiments were determined. Those transcripts with Student’s t-test *p* ≤0.001 and difference of 2-fold or more in expression at 9 and/or 18hpi were considered for analysis. Duplicated Ensembl gene ids (ENSGs) were removed and Ensembl transcript ids (ENSTs) were obtained (Biomart Converter program [http://www.biomart.org/index.html]).

Next, ENSTs were submitted to Genomatix Software Suite Gene2Promoter program (http://www.genomatix.de). This program assigns the accession number to a gene locus in Genomatix’ ElDorado database which relies on *Homo sapiens* GRChr38 build and contains both predicted and experimentally validated promoter sequences. Searches were performed with the following criteria: 1) *Homo sapiens* promoters were analyzed, 2) vertebrate Smad family matrices were to be applied (V$SMAD), and 3) Smad transcription factor response elements needed to be within 1000bp of a defined transcriptional start site. There are five Smad family matrices, V$SMAD.01, V$GC_SBE.01, V$SMAD3.01, V$SMAD3.02 and V$SMAD4.01 (Matrix Library 9.3 March 2015) with a matrix length varying from 8 to 11 base pairs. The output list was further filtered to focus on those ENSTs that had a Smad matrix similarity of ≥ 0.995 (S1 Table).

ENSGs identifiers and the average fold change values for the 913 transcripts that contained predicted Smad response elements within their promoters were imported into Ingenuity Pathway Analysis (IPA, Qiagen Bioinformatics; https://www.qiagenbioinformatics.com/products/ingenuity-pathway-analysis) to determine which cellular networks are populated by these transcripts. Only 898 could be mapped. A comparative analysis of all datasets was performed using a 1.5-fold cutoff to identify canonical pathways that could be related to RVFV pathology to focus on for chromatin immunoprecipitation.

### Chromatin immunoprecipitation (ChIP)

To confirm presence of Smad transcription factors on the predicted promoters ChIP was performed as described [30]. Lysates for immunoprecipitation were generated from mock (media alone), TGF-b activated (100ng/uL for 2hrs), or MP12 infected (MOI 5, 18hpi) HSAEC cells. Primary antibodies (10ug) utilized for immunoprecipitation were Smad4 (R&D Systems # AF2097), tri-methyl-Histone H3 Lysine 4 (mH3K4; CST #9751) and V5 epitope tag (BioRad # MCA1360). Quantitative PCR was performed using SYBR Green PCR Master Mix (Applied Biosystems, 43 09155) with 5 μL of 1:4 diluted immunoprecipitated material and 0.2 μM of primers. Primers were designed to amplify the predicted Smad consensus binding sequence identified in the Gene2Promoter program COL6A3, IL1R2, SEMA6A, SEMA7A, SLIT3, SRGAP3, UNC5A and VAV3. All of the putative promoter sequences provided by Gene2Promoter were cross-referenced against the NCBI Genbank tools to ensure a match to the 5’ transcript. The V5 epitope tag was utilized as background signal. The average Ct value of the V5 immunoprecipitation was calculated for each lysate and primer set in an experiment. This background average was then subtracted from the Ct values for Smad4 and mH3K4 of the same condition to normalize for background. The fold enrichment was then calculated as 2^-ΔΔCt^.

### Statistical analysis

All graphs were plotted using Prism software. Two-way ANOVA statistical analyses were performed using Bonferroni correction for multiple comparisons.

## Results

### RPPA reveals increased phosphorylation of Smad transcription factors

**Fig 1:**
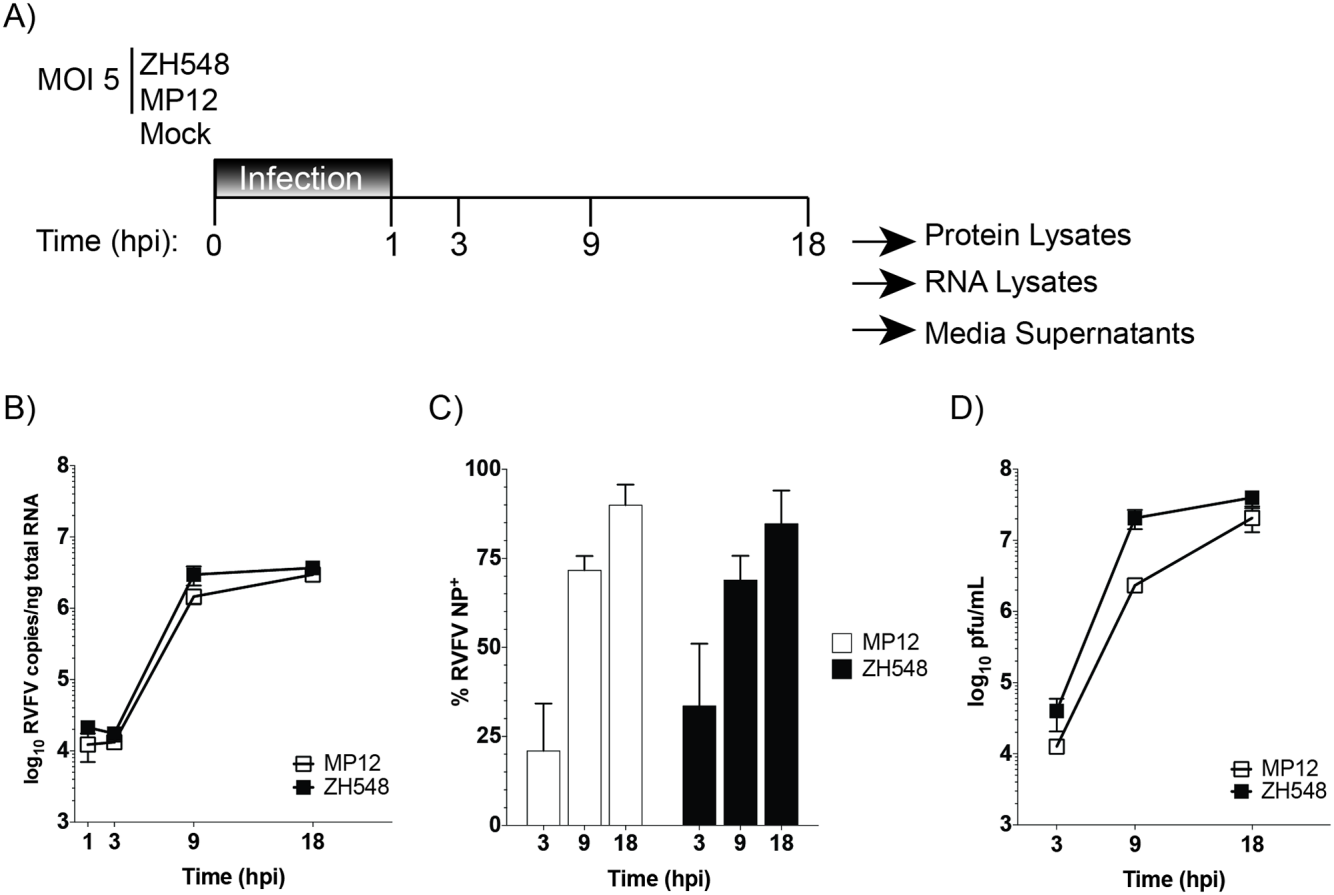
Viral kinetics of virulent and attenuated RVFV viruses. A) Schematic depicting infection and harvest conditions for comparative analysis. HSAECs were infected with attenuated (MP12) or virulent (ZH548) RVFV at an MOI 5 using conditioned media. RVFV RNA replication (B), percentage of RVFV NP positivity (C), and infectious viral titers (D) were determined at the indicated time points (hours post infection (hpi)). The data are plotted as means with standard deviations: for panel (B) n = 3, (C) n = 6 and (D) n = 9. ** = P≤0.01 and **** = P≤0.0001.

To provide a comparative analysis of host responses associated with pathogenesis, a related pair of RVFV strains was used for this study. ZH548, is the parental virulent strain isolated from the 1977–78 RVFV outbreak in Egypt [25, 31]. MP12 is the attenuated, live vaccine candidate generated from serial *in vitro* passaging of ZH548 in the presence of 5-fluoruracil [32]. As a result, MP12 contains a total of 23 nucleotide substitutions (9 nonsynonymous) throughout all three viral segments [33]. For this study, a high multiplicity of infection (MOI) was utilized to minimize signatures detected from uninfected cells during the course of infection and differences in replication kinetics between the virulent and attenuated RVFV strains. Human small airway epithelial cells (HSAECs), a primary immortalized cell line, were chosen because of their utility as a model for aerosol RVFV exposure [34]. To represent early, mid, and late stages of infection, 1, 3, 9, and 18 hours post-infection (hpi) time points were chosen for analysis (Fig 1A). Replication kinetics as measured by viral RNA levels, percentage of RVFV infected cells (*i*.*e*. RVFV NP positivity), and infectious titers (Fig 1B–1D, S1 Fig) demonstrated that MP12 reached levels comparable to the parental virus, ZH548.

**Fig 2:**
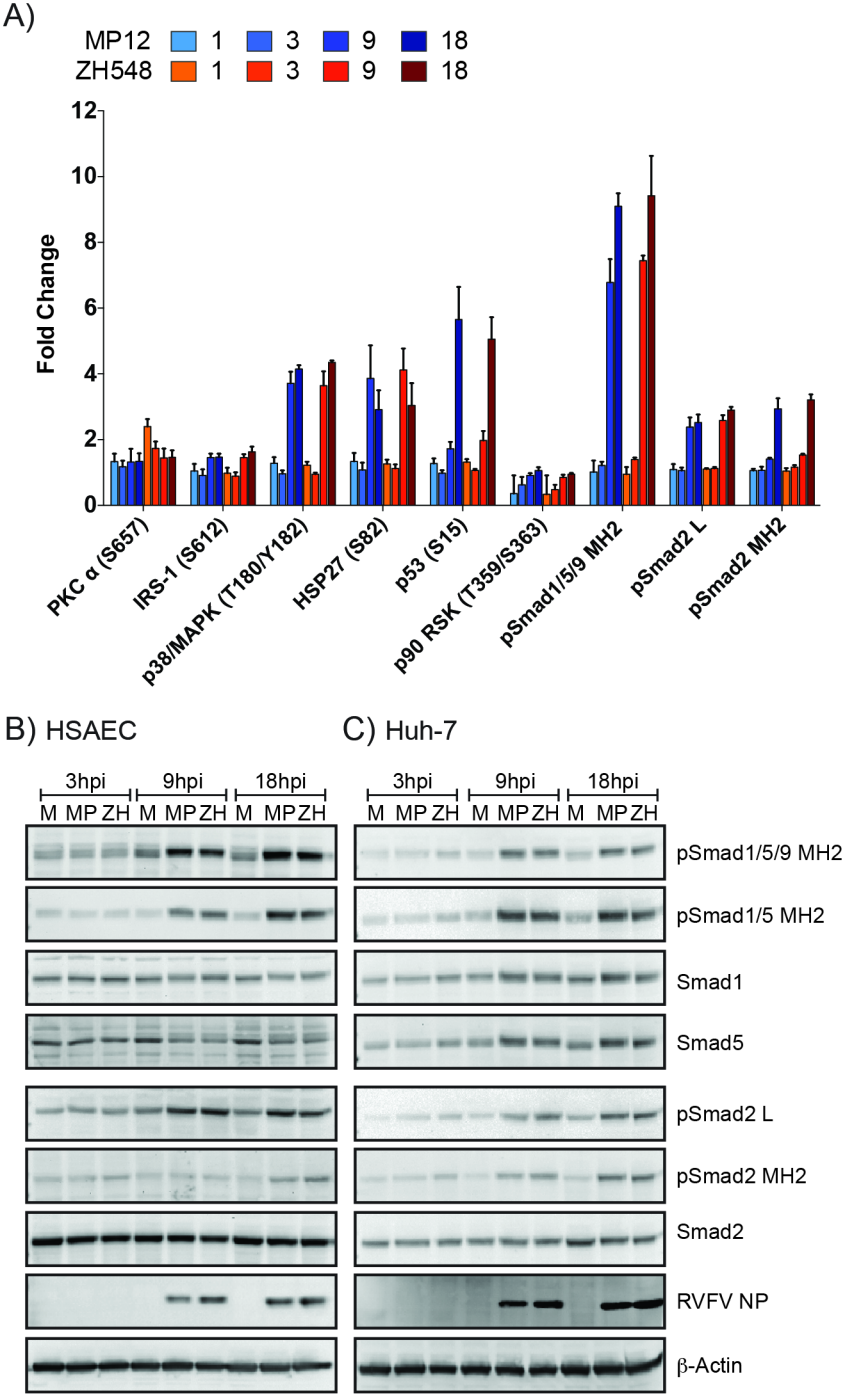
RPPA and Western blot confirmation of Smad protein phosphorylation. A) Phosphorylation status of nine RPPA targets after RVFV infection of HSAECs. Bar graph represents the mean fold change with standard deviation of three independent replicates. Phospho-specific Smad antibodies are noted as: pSmad1/5/9 MH2 [phosphorylated Smad1/5 (S463/465)/9 (S465/467)], pSmad1/5 MH2 [phosphorylated Smad1/5 (S463/465)], pSmad2 MH2 [phosphorylated Smad2 (S465/467)], and pSmad2 L [phosphorylated Smad2 (S245/250/255)]. Statistical significance of the phosphorylation changes are highlighted in Table 1. HSAEC (B) and Huh-7 cells (C) were infected at an MOI 5 with MP12 or ZH548, or mock infected (media alone). Protein lysates harvested at 3, 9, and 18hpi were analyzed for pSmad1/5/9 MH2, pSmad1/5 MH2, pSmad2 MH2, pSmad2 L, RVFV NP, Actin as well as total protein levels for Smad1, Smad5, and Smad2 by western blotting. Blots represent one replicate of three independent experiments. M:Mock; MP:MP12; ZH: ZH548.

The phosphorylation status or overall protein level for 113 select targets was analyzed by RPPA [35]. The protein targets were selected to span a number of signal transduction pathways that influence processes that are known to be or are likely to be influenced by RVFV replication, including cell cycle, apoptosis, growth, inflammation, transcription, translation, autophagy, and innate immune responses. Fold changes were calculated by comparing the signal obtained from RVFV- to mock-infected cell lysates at each corresponding time point (Table 1). The increase in phospho-signaling of several proteins including HSP27 (S82), IRS-1 (S612), p38/MAPK (T180/Y182), p53 (S15), and p90RSK (S380) during RVFV infection mirrored prior analysis with ZH501, thus indicating a strong correlation between differing RVFV strains to alter similar host signaling pathways [20]. Overall, a comparable increase over time in phosphoprotein signaling for both attenuated and virulent RVFV viruses was observed (Fig 2A, Table 1). Only one protein, PKC alpha (S657) showed an initial increase at 1 and 3 hpi in ZH548-, but not in MP12-infected cells. Overall these results suggest that the majority of these host proteins are similarly regulated by both ZH548 and MP12 strains.

**Table 1:** Summary of RPPA results.

		Fold Change^b^											
		MP12 vs Mock				ZH548 vs Mock				ZH548 vs MP12			
Description	Catalog #^a^	1h	3h	9h	18h	1h	3h	9h	18h	1h	3h	9h	18h
**4EBP1 S65**	9451	0.91	0.98	0.86	0.88	**0.81**	0.93	0.80	0.84	0.89	0.95	0.93	0.95
**A-Raf S299**	4431	0.96	0.89	1.12	0.93	1.00	1.09	1.26	1.01	1.04	**1.23**	1.13	1.09
**Acetyl CoA S79**	3661	1.79	1.02	0.83	0.46	1.68	1.08	0.78	0.45	0.94	1.06	0.95	0.97
**Akt S473**	4058	**0.51**	0.76	1.17	1.41	**0.41**	0.75	1.24	1.50	0.80	0.99	1.06	1.07
**Akt T308**	9275	0.84	0.92	**0.72**	**0.64**	0.93	0.90	**0.71**	**0.66**	1.10	0.97	0.98	1.03
**ALK Y1586**	3348	0.98	0.98	1.02	0.96	0.97	1.02	1.05	1.01	0.98	1.05	1.03	1.05
**ALK Y1604**	3341	0.00	0.51	**0.66**	0.97	0.00	0.72	**0.58**	1.02	1.00	**1.41**	0.87	1.05
**AMPKα T172**	4188	1.26	1.07	0.96	0.83	1.19	1.14	1.01	0.86	0.95	1.06	1.05	1.03
**AMPKβ1 S108**	4181	1.11	0.98	1.09	1.10	1.05	1.07	**1.21**	1.18	0.95	1.09	1.11	1.07
**ASK1 S83**	3761	1.19	0.99	1.30	1.01	1.10	1.23	**1.31**	1.06	0.93	**1.24**	1.01	1.05
**ATG5**	2630	0.00	0.79	0.89	0.90	0.00	**0.34**	**0.73**	0.86	1.00	**0.43**	0.82	0.95
**Aurora A-T288/B-T232/C-T198**	2914	1.00	0.98	**0.64**	**0.28**	0.98	0.92	**0.56**	**0.31**	0.98	0.93	0.88	1.12
**B Raf S445**	2696	0.99	1.02	1.02	0.97	1.00	1.11	1.00	1.00	1.01	1.09	0.98	1.04
**BAD S112**	9291	0.62	0.81	1.35	1.15	0.06	**0.60**	**1.39**	**1.28**	**0.09**	**0.74**	1.02	1.11
**BAD S136**	9295	1.00	1.05	**1.49**	**1.59**	0.99	**1.22**	**1.49**	**1.66**	1.00	1.16	1.00	1.04
**Bcl2 S70**	2827	1.01	1.04	1.03	0.94	1.02	1.06	1.05	0.95	1.02	1.02	1.02	1.01
**Beclin 1**	PRS3613	1.02	1.00	1.03	0.81	1.07	1.10	1.06	**0.80**	1.05	1.10	1.03	0.99
**Beta Catenin S33/37/T41**	9561	1.03	1.00	**1.46**	1.02	1.03	0.98	**1.59**	1.09	0.99	0.98	1.09	1.06
**ckit Y703**	3073	1.05	1.08	1.04	0.86	1.04	1.12	1.11	0.91	0.99	1.04	1.07	1.06
**Chk1 S345**	2341	1.06	1.07	1.13	0.89	0.99	1.14	**1.24**	0.80	0.94	1.07	1.10	0.90
*Cleaved Caspase 3 D175	9661	0.00	1.20	1.31	1.28	0.00	0.70	1.64	**1.84**	1.00	0.59	1.26	**1.43**
*Cleaved Caspase 6 D162	9761	0.89	0.94	1.05	1.10	0.79	0.91	1.03	1.03	0.89	0.97	0.99	0.94
*Cleaved Caspase 7 D198	9491	1.11	0.93	1.04	**1.42**	0.96	0.98	0.99	**1.26**	0.87	1.06	0.96	0.89
**Cleaved Caspase 9 D315**	9505	0.87	0.94	0.99	0.97	**0.76**	0.90	0.98	0.82	0.87	0.96	0.98	0.85
**Cleaved PARP D214**	9541	1.13	1.03	1.05	**1.69**	0.94	1.04	1.13	**1.61**	0.83	1.01	1.08	0.95
**Cofilin S3**	3313	0.93	0.84	**1.41**	**1.62**	1.00	1.00	**1.60**	**1.85**	1.07	1.18	1.13	1.14
**cPLA2 S505**	2831	1.07	1.05	1.11	0.90	1.04	1.07	1.16	0.93	0.97	1.02	1.04	1.03
**cRaf S338**	9427	1.07	1.01	1.04	0.87	1.02	1.07	1.11	0.88	0.96	1.06	1.07	1.01
**Cyclin D1 T286**	2921	1.17	0.97	0.88	**0.82**	1.02	0.89	0.81	0.79	0.87	0.92	0.92	0.96
**EGFR Y1068**	2236	1.09	1.00	1.10	**2.93**	1.11	1.02	1.11	**2.82**	1.01	1.02	1.01	0.96
**EGFR Y1148**	4404	0.95	0.94	0.92	0.94	0.96	0.97	0.91	0.95	1.02	1.03	0.99	1.01
**EGFR Y1173**	4407	0.95	0.96	0.92	1.24	0.92	0.94	0.89	1.24	0.97	0.98	0.96	1.00
**EGFR Y992**	2235	1.02	1.08	1.12	1.07	0.92	1.07	1.12	1.09	0.90	0.99	1.00	1.02
**elF4E S209**	9741	1.00	1.13	**1.52**	**2.11**	0.98	**1.34**	**1.62**	**2.34**	0.97	1.18	1.06	1.11
**elF4G S1108**	2441	1.11	1.06	1.05	0.90	1.04	1.08	1.06	0.95	0.94	1.02	1.01	1.06
**Elk1 S383**	9181	0.91	0.91	1.10	1.17	0.88	0.84	1.16	1.14	0.97	0.92	1.06	0.97
**ErbB2 Y1248**	2247	1.00	1.00	0.97	**1.44**	1.01	0.99	0.92	**1.43**	1.01	0.99	0.95	0.99
**ErbB3 Y1289**	4791	0.94	1.00	0.94	0.84	0.96	1.06	0.95	0.89	1.02	1.05	1.02	1.06
**ERK 1/2 T202/Y204**	9101	0.63	0.48	**2.67**	**5.60**	0.46	0.72	3.56	**6.33**	0.73	1.48	1.34	1.13
**FADD S194**	2781	1.08	1.03	**0.61**	**0.22**	1.05	1.00	**0.58**	**0.23**	0.98	0.97	0.95	1.05
**GSK-3β S9**	9336	**0.77**	**0.81**	1.03	**1.22**	**0.67**	**0.74**	0.96	1.20	0.87	0.91	0.93	0.99
**Histone H3 S10**	06–570	0.90	0.83	**0.38**	**0.22**	0.79	0.77	**0.33**	**0.19**	0.88	0.93	0.87	0.89
**Histone H3 S28**	07–145	0.97	0.98	**0.73**	**0.48**	0.92	1.02	**0.75**	**0.47**	0.95	1.04	1.02	0.99
*HSP27 S82	2406	1.32	1.04	**3.75**	**2.81**	1.26	1.11	**4.05**	**2.89**	0.96	1.07	1.08	1.03
**HSP90a T5/7**	3488	1.03	1.00	1.02	**1.37**	1.02	1.06	1.00	**1.23**	0.99	1.05	0.99	0.89
**IGF1 Rec Y1131/Insulin Rec Y1146**	3021	1.07	0.94	0.90	**0.81**	1.01	0.99	**0.82**	**0.70**	0.94	1.05	0.92	0.87
**IGF1R Y1135/36_IR Y1150/51**	3024	0.99	0.96	0.97	**1.78**	1.03	0.92	0.97	**1.89**	1.04	0.97	0.99	1.07
**IkappaB alpha S32/36**	9246	1.08	0.96	0.92	0.97	1.18	0.99	0.94	0.98	1.10	1.03	1.02	1.01
**IL-10**	ab52909	0.99	0.98	0.94	1.05	0.90	1.03	0.93	1.00	0.91	1.05	0.99	0.95
**IL-6**	5143–100	1.00	1.00	1.00	0.97	0.98	1.07	1.00	0.93	0.98	1.07	0.99	0.96
**IL-8**	ab7747	0.95	0.97	0.97	0.99	**0.81**	1.03	0.98	0.94	0.85	1.07	1.01	0.95
**iNOS**	2977	1.06	0.92	0.89	0.89	1.03	0.95	0.83	**0.77**	0.98	1.03	0.93	0.86
*IRS-1 S612	2386	1.03	0.92	**1.45**	**1.46**	0.96	0.89	**1.45**	**1.63**	0.94	0.97	1.00	1.11
**Jak1 Y1022/1023**	3331	0.92	1.04	1.01	1.07	0.86	1.12	1.02	1.07	0.94	1.07	1.01	1.00
**Jak2 Y1007/1008**	3771	0.91	0.98	0.91	0.80	0.86	1.00	0.94	0.81	0.94	1.02	1.04	1.01
*LC3B	3868	1.08	0.95	**0.69**	**0.34**	1.06	0.96	**0.74**	**0.37**	0.98	1.01	1.07	1.09
**LIMK1 T508/LIMK2 T505**	3841	1.05	0.91	0.93	0.89	1.07	0.94	0.86	0.85	1.02	1.03	0.92	0.95
**MARCKS S152/156**	2741	1.09	0.99	0.91	**0.65**	1.08	1.02	0.84	**0.62**	0.99	1.03	0.93	0.95
**MDM2 S166**	3521	1.06	0.94	0.90	0.76	0.97	0.96	0.87	**0.73**	0.92	1.03	0.97	0.95
**MEK 1–2 S217/221**	9121	1.03	1.03	0.86	**0.62**	0.99	1.01	0.86	**0.61**	0.96	0.98	1.00	0.99
**Met Y1234/1235**	3126	1.01	0.94	0.92	0.77	1.04	1.01	0.93	**0.79**	1.04	1.07	1.01	1.03
**MMP-11**	ab52904	0.99	0.95	0.91	0.90	1.19	0.93	0.85	**0.79**	1.20	0.97	0.93	0.88
**MMP-9**	3852	1.07	1.01	1.06	1.09	1.01	1.03	1.15	1.14	0.94	1.02	1.09	1.05
**MPO**	4162	1.11	0.98	0.96	1.01	1.05	1.11	1.02	1.03	0.94	1.13	1.06	1.01
**MSK1 S360**	9594	0.87	1.01	1.13	**1.29**	**0.69**	1.04	**1.28**	**1.30**	**0.79**	1.03	1.14	1.01
**Mst1 T183/Mst2 T180**	3681	0.95	0.89	0.88	0.90	0.88	0.85	**0.81**	0.84	0.93	0.95	0.92	0.93
**mTOR S2448**	2971	1.00	0.95	1.06	1.02	0.94	0.97	1.12	1.04	0.93	1.02	1.05	1.02
*NFkB p65 S536	3031	1.08	1.03	**2.36**	1.22	1.12	1.05	**2.55**	**1.22**	1.05	1.02	1.08	1.00
**NPM T199**	3541	0.99	1.00	0.90	**0.82**	0.96	1.01	0.92	0.85	0.97	1.02	1.03	1.03
**p27 T187**	71–7700	1.03	0.93	**0.71**	**0.62**	1.07	0.89	**0.67**	**0.62**	1.05	0.96	0.95	0.99
*p38/MAPK T180/Y182	9211	1.27	0.95	**3.69**	**4.14**	1.23	0.95	**3.65**	**4.35**	0.97	1.00	0.99	1.05
*p53 S15	9284	**1.27**	0.98	**1.71**	**5.67**	**1.32**	1.06	**1.95**	**5.08**	1.04	1.09	1.14	0.90
**p70S6 T389**	9205	0.97	0.82	1.19	1.08	0.74	0.75	1.02	1.14	0.76	0.91	0.86	1.05
**p90 RSK T359/S363**	9344	0.05	**0.62**	0.91	1.05	0.00	**0.48**	0.85	0.94	0.00	0.77	0.93	0.90
*p90RSK S380	9341	1.16	0.94	**1.28**	**1.42**	**1.23**	0.90	**1.28**	**1.43**	1.05	0.96	1.00	1.01
**PDGFR Y716**	07–021	0.97	1.01	0.99	1.16	0.99	1.03	0.99	1.18	1.02	1.02	1.00	1.01
**PDGFR Y751**	3161	**1.29**	1.03	1.01	1.18	**1.26**	1.11	1.09	**1.24**	0.97	1.07	1.08	1.05
**PDK1 S241**	3061	1.08	1.00	0.98	**0.76**	1.09	1.04	1.02	**0.81**	1.01	1.04	1.05	1.08
**PI3K p110**	4252	0.98	1.01	0.97	1.17	0.93	1.01	0.92	1.10	0.96	1.00	0.94	0.94
**PKA C T197**	4781	1.03	0.98	1.04	1.11	1.01	1.06	1.05	1.12	0.98	1.09	1.01	1.01
**PKC alpha S657**	06–822	1.34	1.17	1.27	1.31	**2.40**	**1.72**	**1.40**	**1.43**	**1.79**	**1.47**	1.10	1.09
**PKC delta T505**	9374	1.08	0.96	0.97	0.98	1.12	1.02	0.98	0.94	1.04	1.06	1.01	0.95
**PKC zeta lambda T410/403**	9378	1.06	0.96	1.13	1.11	0.97	1.00	1.07	1.12	0.92	1.04	0.95	1.00
**PLCγ1**	2821	1.04	1.00	1.04	0.91	1.07	1.06	1.09	0.97	1.03	1.06	1.05	1.07
**Protein Phosphatase 1 Beta**	ab53315	1.01	0.97	0.98	1.12	1.01	0.97	0.97	1.13	1.01	0.99	0.99	1.01
**PTEN S380**	9551	0.98	0.98	1.00	0.95	0.99	1.05	1.02	0.97	1.01	1.07	1.02	1.02
**Raf S259**	9421	1.02	0.96	1.02	0.85	0.99	1.03	1.05	0.86	0.97	1.07	1.04	1.02
**Ras/GRF1 S916**	3321	0.94	1.01	0.96	**0.81**	0.87	1.00	0.96	**0.80**	0.92	0.99	1.00	0.99
**Rb S780**	3590	1.02	0.94	0.71	**0.43**	0.98	0.94	0.76	**0.44**	0.96	1.00	1.06	1.03
**Ron Y1353**	5176–1	1.07	1.04	1.03	0.80	1.02	1.11	0.99	**0.80**	0.95	1.07	0.96	0.99
**S100A7 Calcium BP**	H00006278-A01	1.04	0.97	0.95	1.17	1.04	1.06	0.88	1.00	0.99	1.08	0.93	0.86
**S6 Ribosomal Protein S235/236**	4856	1.15	0.99	1.56	0.99	0.84	0.85	**1.66**	1.15	**0.73**	0.85	1.06	1.16
**SAPK/JNK T183/Y185**	9251	1.09	0.90	**1.64**	**1.63**	1.09	**0.82**	**1.56**	**1.65**	1.01	0.91	0.95	1.02
**SEK1/MMK4 S80**	9155	0.96	0.96	1.06	1.16	0.89	0.96	1.06	1.17	0.92	1.01	1.00	1.01
**SHIP1 Y1020**	3941	0.96	0.92	0.94	0.75	1.02	1.03	1.01	0.84	1.06	1.12	1.07	1.12
**Smad1/5 (Ser463/465)/ Smad9 (Ser465/467)**	9511	0.95	1.21	**6.75**	**9.07**	0.91	1.40	**7.44**	**9.36**	0.95	1.15	1.10	1.03
**Smad2 S245/250/255**	3104	1.07	1.05	**2.37**	**2.51**	1.09	1.12	**2.57**	**2.89**	1.02	1.07	1.09	1.15
**Smad2 S46/467**	3101	1.05	1.06	**1.41**	**2.93**	1.04	1.16	**1.53**	**3.21**	0.99	1.09	1.09	1.10
**SOCS1**	3950	1.00	1.11	1.22	0.83	1.09	1.29	1.34	0.99	1.10	1.16	1.10	1.20
**SOCS3**	2923	0.88	0.99	0.84	0.65	0.93	0.87	0.73	0.71	1.06	0.89	0.87	1.09
**Src Family Y416**	2101	0.95	1.06	1.04	1.11	0.91	1.09	1.00	1.20	0.95	1.03	0.97	1.09
**Src Y527**	2105	0.99	0.93	0.92	0.85	1.01	0.96	0.93	0.88	1.01	1.03	1.00	1.04
**Stat1 Y701**	9171	1.01	0.98	1.07	**1.30**	0.98	1.00	1.04	1.10	0.97	1.01	0.97	0.85
**Stat3 S727**	9134	1.03	0.95	**1.25**	1.05	0.99	0.98	**1.30**	1.17	0.96	1.02	1.04	1.11
**Stat3 Y705**	9145	1.02	0.93	**1.32**	2.32	0.96	0.85	**1.50**	**1.88**	0.94	0.91	1.14	0.81
**Stat4 Y693**	5267	1.03	0.86	0.97	1.06	0.98	0.90	0.95	1.01	0.95	1.05	0.97	0.95
**Stat5 Y694**	9351	1.02	0.99	0.99	**0.63**	1.02	1.01	0.92	**0.60**	1.00	1.01	0.94	0.95
**Stat6 Y641**	9361	1.01	0.86	1.07	**1.33**	0.93	0.86	1.02	**1.21**	0.92	1.00	0.95	0.91
**Ubiquitin**	3936	1.16	0.93	0.76	0.75	1.21	1.00	0.82	0.78	1.05	1.07	1.08	1.04
**VASP S157**	3111	1.02	0.98	1.18	1.19	0.98	0.98	1.14	**1.26**	0.97	1.00	0.96	1.06
**VEGFR2 Y1175**	2478	1.00	1.05	0.99	0.99	0.98	1.12	0.98	0.95	0.98	1.07	1.00	0.96
**VEGFR2 Y996**	2474	0.99	1.00	1.00	**0.69**	0.98	1.07	0.98	**0.71**	1.00	1.07	0.98	1.02
**Wnt5 a/b**	2530	1.00	1.02	0.97	0.91	1.01	1.10	0.97	0.97	1.00	1.09	0.99	1.06

* Protein names with asterisks correlate with ZH501 trends [20].

^a^Unless noted otherwise within Experimental Procedures all antibodies are from Cell Signaling

^b^Values highlighted and bolded represent 1.2 difference in fold change and T-test, P<0.05. Increased fold change values are highlighted in blue and decreased in yellow.

One of the most highly phosphorylated proteins identified from this targeted screen were the R-Smad transcription factors that are phosphorylated by and transducers of the TGF-β superfamily mediated signaling. Increased phosphorylation of the MH2 domain containing serine residues, which correspond to receptor mediated activation [36, 37], for both Smad1/5/9 and Smad2 occurred as early as 9 hpi. By 18hpi, a 9- and 3-fold induction over mock for phosphorylated Smad1/5/9 and Smad2, respectively, was observed (Fig 2A). Phosphorylation of serine residues located within the linker region of Smad2, which are MAPK-dependent [38–40], were also increased more than 2-fold at 9 and 18hpi. Western blot analysis of RVFV infected HSAECs and a hepatoma cell line, Huh-7, confirmed increased phosphorylation of these R-Smads (Fig 2B and 2C). While total Smad2 protein levels remained constant in both HSAECs and Huh7 cells, some cell type differences were observed for total Smad1 and -5. In HSAECs Smad1 and -5 protein levels were slightly reduced and a slower migrating form was detected at 9 and 18hpi (Fig 2B). In Huh7 cells at 9 and 18 hpi, Smad 1 and -5 protein levels were increased and similarity had a lower migrating form (Fig 2C). Levels of Smad transcripts were assessed by RT-qPCR for RVFV infections of HSAEC and Huh-7 cells (S2 Fig). In both cell backgrounds a decrease in relative RNA levels was observed for all Smad transcripts by 18hpi.

**Fig 3:**
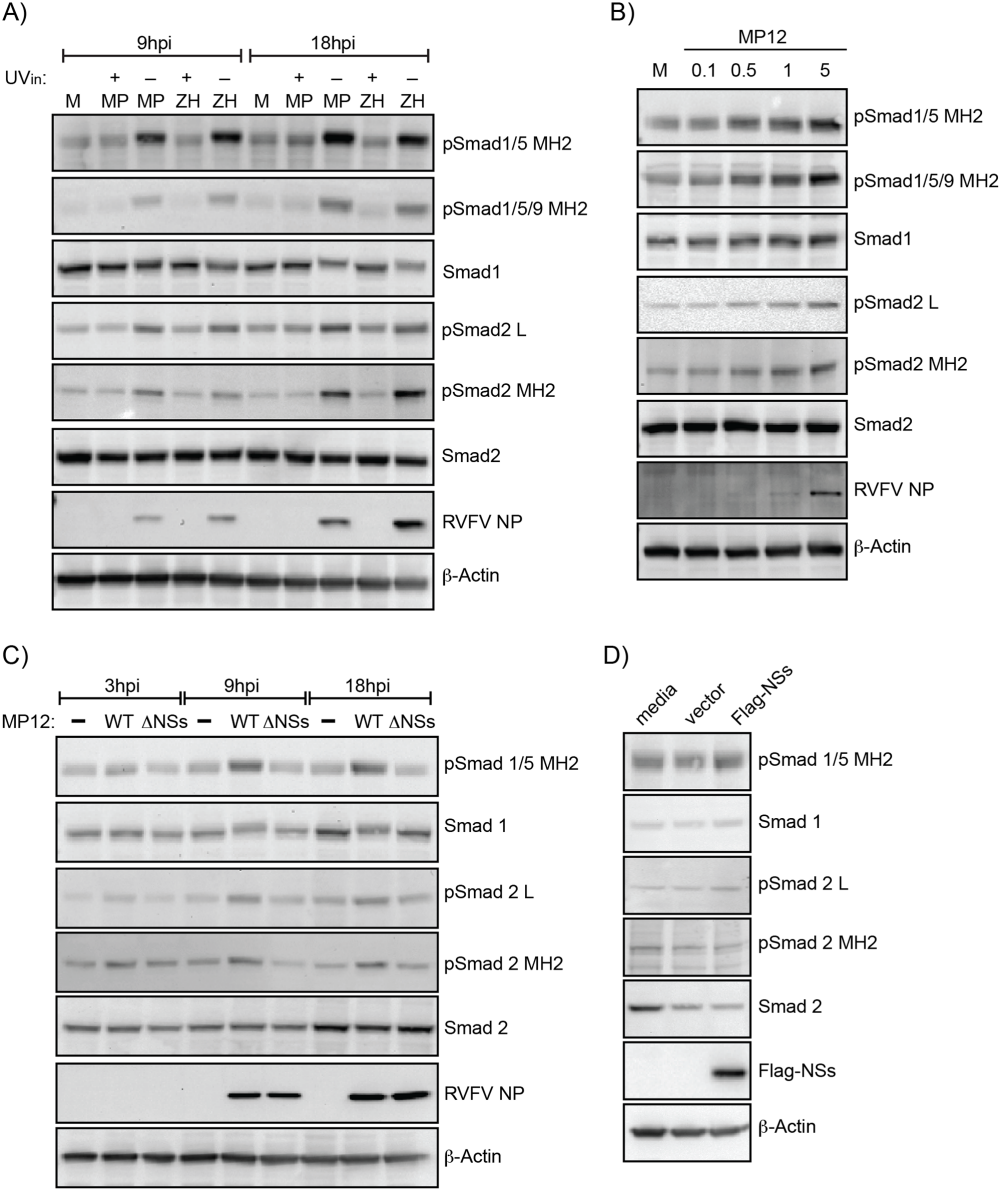
Smad phosphorylation is dependent on RVFV replication and NSs expression. A) Analysis of HSAECs infected with MP12, ZH548 or their UV-inactivated (UVin) controls at 9 and 18hpi. Protein lysates were analyzed by western blot for pSmad1/5/9 MH2, pSmad1/5 MH2, pSmad2 MH2, pSmad2 L, RVFV NP, actin as well as total protein levels for Smad1 and Smad2. M: Mock; MP: MP12; ZH: ZH548. B) Smad phosphorylation in HSAECs infected with increasing MOIs (0.1, 0.5, 1, and 5) of MP12 virus at 9hpi was examined as described in panel A. C) Analysis of Vero cells infected at an MOI 3 with MP12 or MP12 ΔNSs. With the exception of pSmad1/5/9 MH2, protein lysates were analyzed by western blot as described in panel A. D) Vero cells transfected with media alone, pcDNA3.1 vector (2.5 μg), or pcDNA3.1-Flag-NSs (2.5 μg) were harvested at 24 hours post transfection. Protein lysates were analyzed by western blot for pSmad1/5 MH2, pSmad2 MH2, pSmad2 L, Flag-NSs as well as total protein levels for Smad1 and Smad2.

Several different conditions (inactivated viruses, differing MOIs, NSs dependency) were evaluated for R-Smad activation. At 9 and 18hpi, HSAEC cells exposed to UV-inactivated MP12 or ZH548 lacked an increase in Smad phospho-signaling indicating that active RVFV replication was required (Fig 3A). Increasing MOIs for MP12 infection correlated with increased levels of phosphorylation within the MH2 domain and linker region (Fig 3B). To determine if Smad activation was NSs-dependent, a recombinant MP12 virus with NSs deleted and an NSs expression construct were both utilized (Fig 3C and 3D). NSs is the main RVFV virulence factor and disrupts interferon signaling by targeting for degradation several proteins involved in transcriptional and translational control, including p62/TFIIH and PKR [24, 41–45]. Loss of NSs expression with this recombinant MP12 virus lacked R-Smad phosphorylation and resembled mock-infected levels, while overexpression of NSs protein alone was not sufficient to increase R-Smad phosphorylation. This suggests that while loss of NSs impairs the activation and/or maintenance of R-Smad signaling, NSs alone is not responsible for initiating this signaling cascade.

### siRNA knockdown of Smad1, 2, and 4 do not impact RVFV replication

**Fig 4:**
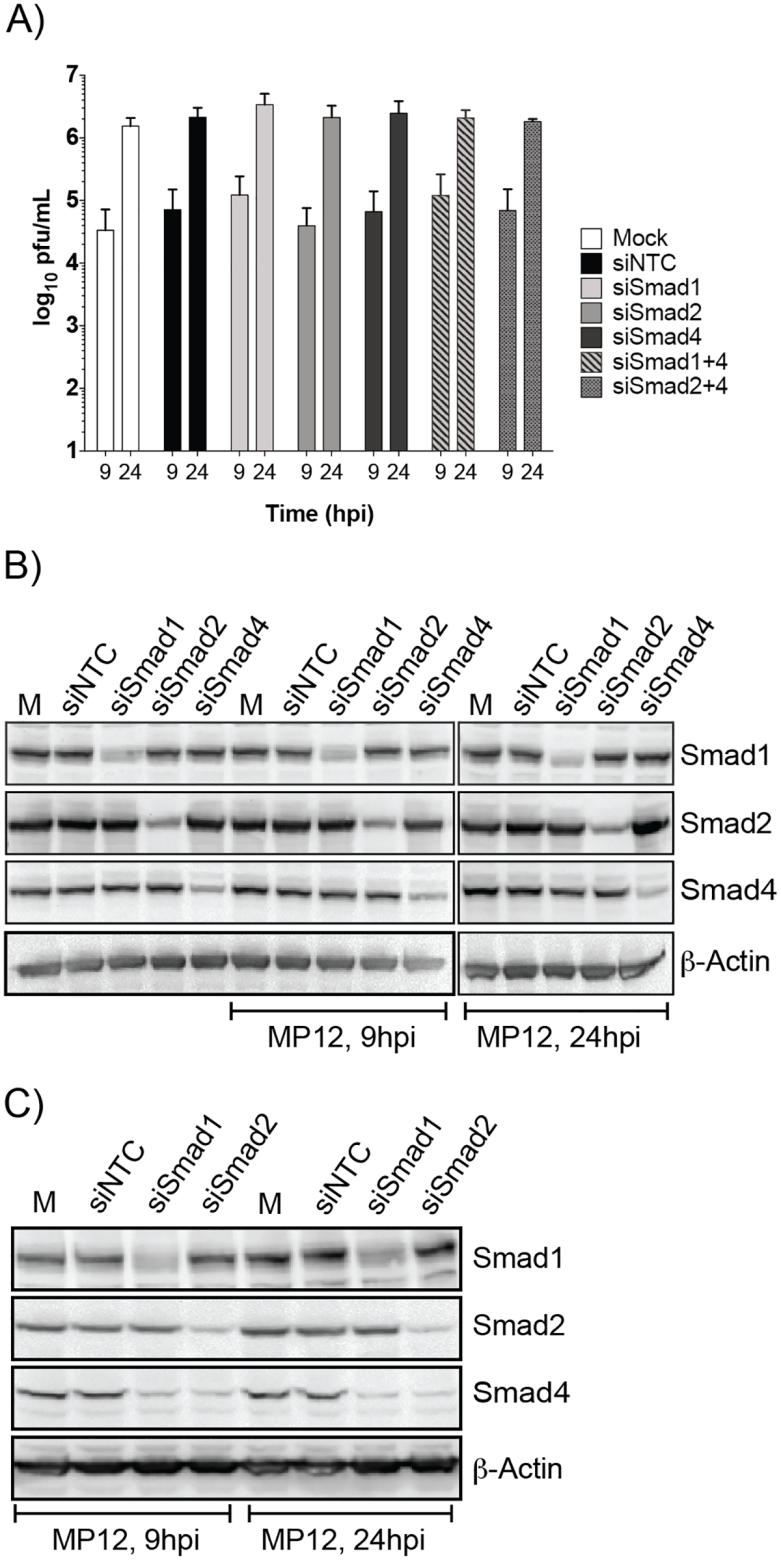
siRNA knockdown of Smad1, -2 and 4 does not impact RVFV replication. HSAECs transfected with media alone (Mock) or siRNAs (75nM) directed against a nontargeting control (NTC), Smad1, -2, or -4 were infected with MP12 virus (MOI 0.1). Both extracellular media supernatants (A) and protein lysates (B,C) were harvested at 9 and 24hpi. A) Infectious viral titers were determined by plaque assay. Data plotted as a bar graph of the mean with standard deviation of four replicates. Protein lysates from single (B) and double knockdowns (C) were analyzed by western blot for actin, total Smad1, -2, and -4 expression.

To determine if Smads directly impacted RVFV replication, knockdown of Smad1 and -2 or Smad4 either alone or in combination was performed (Fig 4). A 75–80% decrease in Smad protein levels was observed (Fig 4B). Infectious MP12 titers were examined at 9 and 24hpi (Fig 4A). Overall there was a slight increase in viral titers at both timepoints for Smad1 alone and at the 9hpi timepoint for the double Smad1/4 knockdown, however this difference was not statistically significant. Therefore, given that no direct correlation could be made between Smad activation on RVFV replication we instead focused on possible contribution to RVFV-mediated pathology.

### Identification of possible Smad-regulated promoters

**Fig 5:**
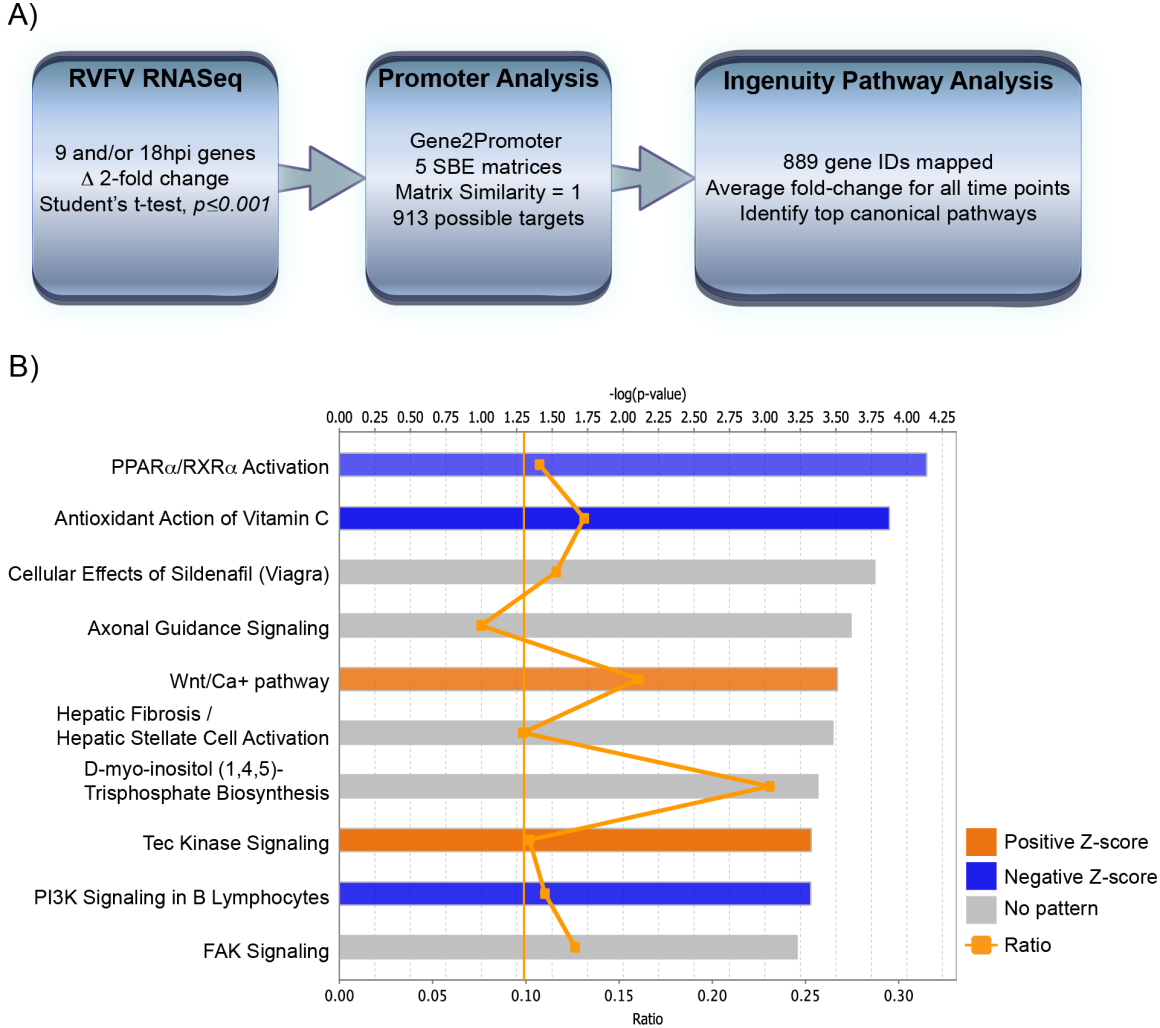
Nuclear localization of phosphorylated Smad proteins. HSAEC cells either alone (Mock), treated with TGF-β3 (50ng/mL, 2hrs), BMP-4 (50ng/mL, 2hrs), or infected with MP12 (MOI 5) for 9 or 18hpi were cell fractionated. 10ug of nuclear extracts (NE) or cytoplasmic extracts (CE) were probed for pSmad1/5 MH2, pSmad 2 MH2, GAPDH and Lamin A/C levels.

R-Smads, whose activation is regulated temporally and spatially, can regulate in conjunction with other transcription factors a complex arrangement of genes involved in development, differentiation, and homeostasis. After translocation into the nucleus, Smad complexes, comprised of dimeric R-Smads associated with Smad4, can bind to DNA at Smad-binding elements with low affinity (as reviewed in [16]). However, the interaction with additional partners, including transcription factors and chromatin modifying enzymes, enhances Smad-binding specificity and affinity. Depending on associated partners, Smad complexes may promote or inhibit transcription from TGF-β/BMP-stimulated promoters. To determine if Smad protein phosphorylation resulted in their nuclear localization, cellular fractionation followed by western blot analysis was performed. Nuclear pSmad1/5 MH2 was observed in mock infected cells and in cells treated with TGF-β and BMP (Fig 5). Little to no nuclear pSmad2 MH2 was present in mock infected cells, but TGF-β induced nuclear accumulation of pSmad2 MH2. Both pSmad1/5 MH2 and pSmad2 MH2 nuclear localization was observed in RVFV MP12 infected cells at 9 and 18 hpi.

**Fig 6:**
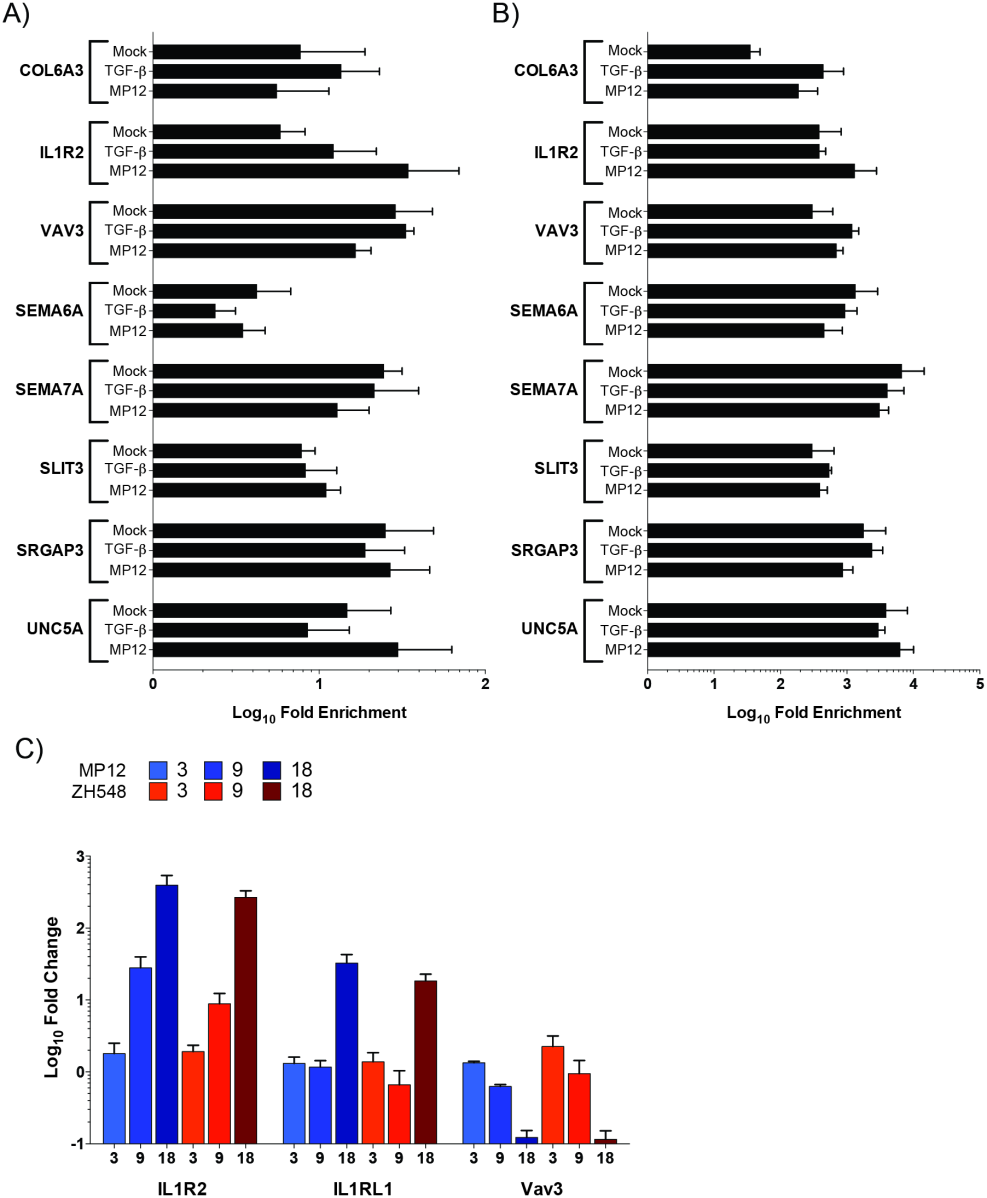
Promoter analysis of RVFV RNASeq yields potential Smad-dependent promoters. A) Schematic depicting data-mining of RVFV RNASeq dataset for Smad-dependent promoters. B) Top ten significantly altered canonical pathways from IPA are shown for the virulent RVFV strain at 18hpi. The top axis indicates the statistical significance calculated using the right-tailed Fisher exact test. Threshold line represents significance cut-off at *p = 0*.*05*. Bars represent the level of significance, with orange and blue bars indicating whether the pathway is predicted to be activated or inhibited, respectively (z-score). Pathways where no prediction can be made are colored gray. The ratio (bottom axis) represents the number of molecules identified in a given pathway divided by total number of molecules that constitute that pathway.

To identify possible Smad-regulated genes differentially expressed after RVFV infection, a promoter analysis was performed (Fig 6A). Using a RVFV RNASeq dataset [29], those transcripts that were altered 2-fold or more at 9 and/or 18hpi were analyzed using Gene2Promoter program [46, 47]. This program contains five validated Smad-binding element matrices, varying in length from 8 to 11 base pairs. Identified genes with matches that contained a sequence similarity of 1 (*i*.*e*. 100% match) were further evaluated by Ingenuity Pathway Analysis (IPA) software [48]. Out of the 913 possible Smad-regulated genes, 889 gene IDs could be mapped and the top ten canonical pathways were identified based on the ZH548 versus mock-infected comparison at 18hpi (Table 2, Fig 6B). These same pathways were also identified in the MP12 dataset although some pathways had additional genes seeded from the virulent strain comparison, *e*.*g*. COPS5, HKR1, ITGA3, NGEF and UNC5A in axonal guidance were ZH548-specific.

**Table 2:** Top ten canonical pathways for possible Smad-regulated genes.

Canonical Pathway	MP12	ZH548	Gene List^a^
	*p-value*	*p-value*	
PPARa/RXRa Activation	3.1E-05	7.2E-05	ACVR1, ADIPOR1, CYP2C18, CYP2C19, GNAS, IL1R2, IL1RL1, JAK2, MAP4K4, MED12, NFKB1, NFKB2, NFKBIA, PLCB4, PLCD4, PLCG2, PLCL1, PRKAB2, SMAD3
Antioxidant Action of Vitamin C	7.0E-05	1.3E-04	JAK2, NFKB1, NFKB2, NFKBIA, PLA2G4C, PLA2R1, PLCB4, PLCD4, PLCG2, PLCL1, PLD6, SLC23A2, SLC2A5
Cellular Effects of Sildenafil (Viagra)	9.7E-04	1.7E-04	ACTC1, CACNA1E, **CACNA1S**, GNAS, GUCY1A2, KCNN1, MYL5, PABPC4, PDE2A, **PDE4B**, PLCB4, PLCD4, PLCG2, PLCL1, SLC4A10
Axonal Guidance Signaling	3.1E-03	2.5E-04	ACE, ADAM20, ARHGEF6, **COPS5**, EFNB2, EPHA4, ERAP2, FYN, GNAS, HERC2, **HKR1**, **ITGA3**, MYL5, MYSM1, NCK1, NFAT5, **NGEF**, NRP2, PAK3, PAK5, PAPPA, PIK3R3, PLCB4, PLCD4, PLCG2, PLCL1, SEMA4A, SEMA6A, SEMA7A, SHANK2, SLIT3, SRGAP3, **UNC5A**
Wnt/Ca+ pathway	1.9E-04	3.1E-04	CREB1, CREB5, NFAT5, NFKB1, NFKB2, PLCB4, PLCD4, PLCG2, PLCL1
Hepatic Fibrosis / Hepatic Stellate Cell Activation	4.5E-04	3.3E-04	COL12A1, COL28A1, **COL5A1**, COL6A3, COL8A1, COL9A2, EGFR, FAS, FN1, IGF2, IL1R2, IL1RL1, LAMA1, MYL5, NFKB1, NFKB2, SMAD3, VCAM1
D-myo-inositol (1,4,5)-Trisphosphate Biosynthesis	2.3E-03	4.2E-04	**PI4KA**, PIP5K1B, PLCB4, PLCD4, PLCG2, PLCH1
Tec Kinase Signaling	7.1E-04	4.7E-04	ACTC1, BMX, FAS, FYN, GNAS, **ITGA3**, JAK2, NFKB1, NFKB2, PAK3, PAK5, PIK3R3, PLCG2, PTK2B, RHOJ, VAV3
PI3K Signaling in B Lymphocytes	2.5E-04	4.8E-04	ATF3, C3, CREB1, FYN, NFAT5, NFKB1, NFKB2, NFKBIA, PLCB4, PLCD4, PLCG2, PLCL1, PLEKHA4, VAV3
FAK Signaling	1.3E-03	5.9E-04	ACTC1, ARHGAP26, ARHGEF6, ASAP1, EGFR, FYN, **ITGA3**, PAK3, PAK5, PIK3R3, PLCG2

^a^Gene names in bold are ZH548-specific

**Fig 7:**
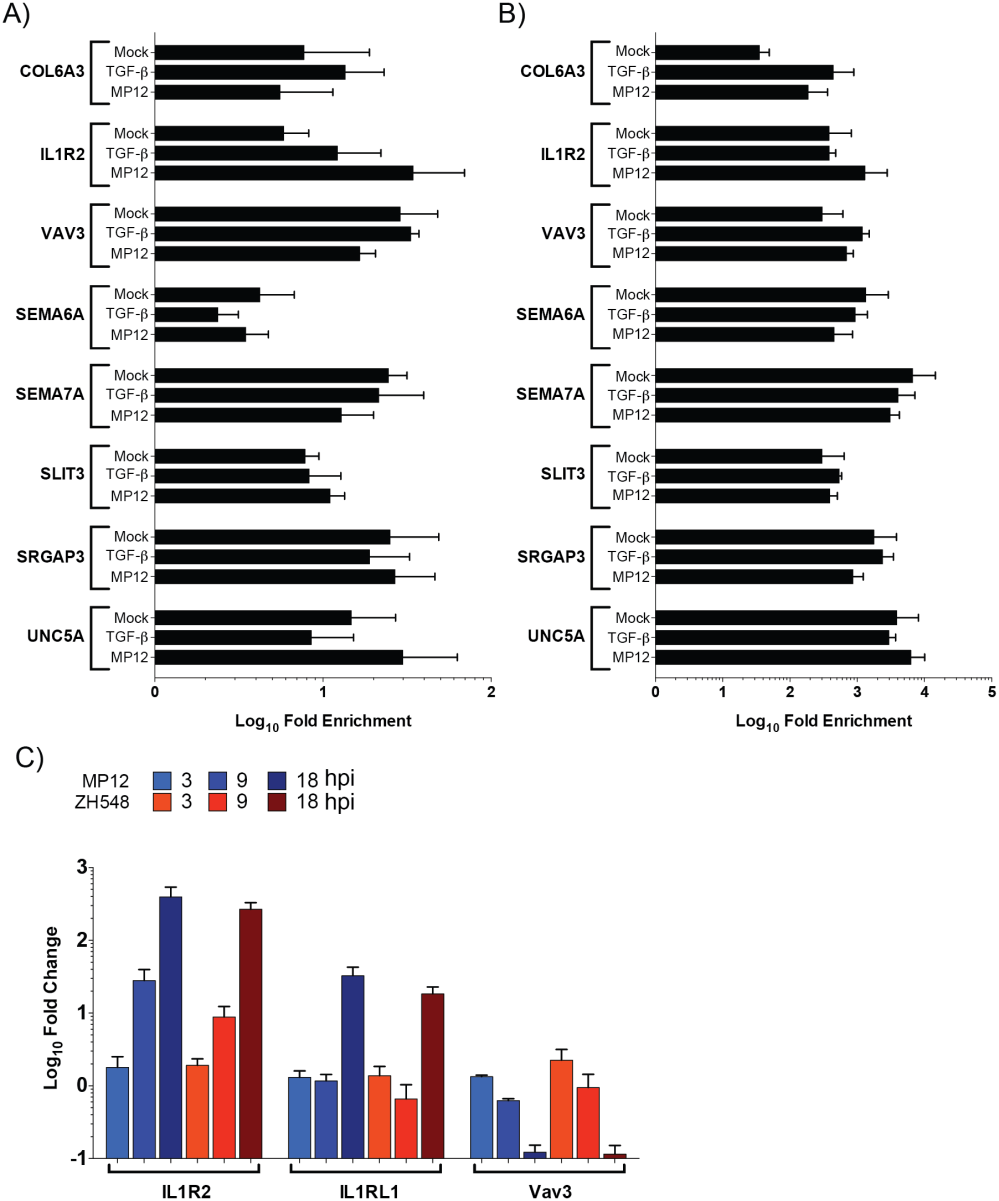
ChIP analysis of possible Smad-dependent transcripts. Lysates from mock, TGF-β activated, and MP12 infected were utilized for Smad4 (A) or methylated histone H3 lysine 4 (mH3K4; B) immunoprecipitations. Relative DNA abundance from immunoprecipitations was determined by qPCR. Signal over background normalization and fold enrichment was calculated for each condition. Bars represent means and standard deviations of four replicates. C) Levels of IL1R2, IL1RL1, and VAV3 RNA are analyzed by qRT-PCR. RNA lysates from HSAECs infected with MP12 (blue) or ZH548 (red) RVFV at an MOI 5 were utilized. Bars represent means and standard deviations of four replicates.

ChIP was utilized to confirm the presence of Smad complexes on the promoters of several transcripts from axonal guidance, hepatic fibrosis/stellate cell activation and Tec kinase signaling pathways (Fig 7A and 7B). These pathways were chosen for various reasons. While critical during development, recent reports have shown that the axonal guidance proteins are necessary for plasticity of mature neuronal tissue. Expression of these proteins has also been shown to be critical for non-neuronal tissue. The liver is a target organ for RVFV replication with the majority of infected humans, ruminants and laboratory animals, such as mice, displaying hepatic necrosis and/or elevated AST/ALT levels. Tec kinases are a large non-receptor tyrosine kinase family that are activated downstream of many cell-surface receptors including G-protein coupled receptors (GPCRs), integrin, antigen, cytokine and TNF receptors. These kinases can regulate numerous cellular processes including, calcium influx, apoptosis, gene expression, actin reorganization and adhesion/migration. Therefore, a small subset of these potential Smad-regulated genes that demonstrated a 2-fold or greater difference by RNASeq were included for ChIP analysis; these targets are COL6A3 and IL1R2 (hepatic fibrosis/stellate cell activation), SEMA6A, SEMA7A, SLIT3, SRGAP3, and UNC5A (axonal guidance), and VAV3 (Tec kinase signaling; Table 3).

**Table 3:** Potential Smad-regulated genes selected for ChIP analysis.

Symbol	Entrez Gene Name	Location	Function	MP12 vs Mock*			Zh548 vs Mock*		
				3h	9h	18h	3h	9h	18h
COL6A3	collagen type VI alpha 3	Extracellular Space	one of the three alpha chains of type VI collagen	-1.2	1.8	3.2	-1.1	2.3	3.7
IL1R2	interleukin 1 receptor type 2	Plasma Membrane	Decoy receptor that inhibits the activity of its ligands (IL1A, IL1B). Interacts with IL1R1.	1.0	3.9	30.4	-1.2	1.9	20.8
SEMA6A	semaphorin 6A	Plasma Membrane	Axon repulsion guidance ligand.	-1.3	1.1	2.3	-1.2	1.6	2.4
SEMA7A	semaphorin 7A	Plasma Membrane	immune semaphorin—integrin-mediated signaling and functions both in regulating cell migration and immune responses.	-1.4	1.4	2.1	-1.1	1.7	2.3
SLIT3	slit guidance ligand 3	Extracellular Space	Interacts with ROBO receptors to negatively direct cell migration—axon guidance	1.2	1.7	2.1	1.3	1.9	3.7
SRGAP3	SLIT-ROBO Rho GTPase activating protein 3	Cytoplasm	negatively regulates cytoskeletal reorganization	1.7	3.8	19.2	1.4	4.4	15.2
UNC5A	unc-5 netrin receptor A	Plasma Membrane	Netrin receptor required for repulsion of growth cone in axon guidance.	-1.1	-1.0	1.4	1.0	1.0	2.1
VAV3	vav guanine nucleotide exchange factor 3	Cytoplasm	interacts with Rho family GTPases that activate pathways leading to actin cytoskeletal rearrangements and transcriptional alterations	-1.3	-1.4	-3.2	-1.1	-1.3	-3.8

*Average RNASeq fold change

Antibodies directed against Smad4, methylated histone 3 lysine 4 (mH3K4), and V5 epitope tag were utilized for our ChIP experiments. Smad4 would be associated with both TGF-β and BMP receptor stimulated R-Smad complexes, while mH3K4 modification serves as a positive control antibody and can be associated with transcriptionally active promoters. Lysates from both mock (media alone), TGF-β stimulated, and MP12-infected HSAEC cells were utilized for immunoprecipitation. While TGF-β treatment primarily activates the Smad2/3 complex, it also cross-activates the Smad1/5/9 complex to a degree [49]. After RVFV infection (compared to mock infected cells), mH3K4 was marginally increased on COL6A3 andIL1R2 promoters (Fig 7B). Out of all eight targets, only IL1R2, an IL1 decoy receptor, demonstrated enrichment for Smad4 after MP12-infection (average 34.4-fold) as compared to the mock control (5.8-fold; Fig 7A), although these results were not statistically significant. Furthermore, TGF-β activation only slightly increased Smad4 presence on the IL1R2 promoter (12.2-fold). Thus while MP12 infection stimulated active transcription on most of these promoters by 18hpi it was only on the IL1R2 promoter that increased Smad4 occupancy was observed. To confirm the trends in mRNA levels of IL1R2 and VAV3 transcripts observed by RNASeq, RT-qPCR was performed (Fig 7B). VAV3 decreased over time after RVFV infection. Conversely, IL1R2 and an additional possible Smad-regulated gene (Table 2), interleukin 1 receptor like 1 (IL1RL1), was observed to increase over time. Therefore, RVFV infection leads to Smad activation and hence, increased presence of Smad complexes on promoters such as interleukin 1 family of receptors.

## Discussion

From our targeted RPPA screen, we did not observe many unique differences between the attenuated and virulent RVFV strains. Only one target, PKCα S657, demonstrated higher level of phosphorylation during early ZH548 infection as compared to MP12 (1hpi 1.79-fold and 3hpi 1.47-fold, Table 1). PKC is a calcium-activated, phospholipid- and diacylglycerol (DAG)-dependent serine/threonine-protein kinase that is upstream of several signaling cascades including p38/MAPK [50]. Many viruses utilize PKC signaling during entry, viral fusion or later steps within the lifecycle, including HIV, IAV and HSV [51–53]. A prior report had shown that the ε isoform and not the classical PKC kinases (α,β, or γ) was required for MP12 viral entry in both mammalian and *Drosophila* cells [54]. Additionally, lower phosphorylation of the BAD S112 in ZH548 was also observed (1hpi 0.09-fold and 3hpi 0.74, Table 1). Phosphorylation of BAD, a pro-apoptotic protein, alters its association from survival protein, BSL-X_L_ and BSL-2, to 14-3-3 scaffolding proteins [55, 56]. This release from BAD’s negative inhibition of these two proteins leads to cell survival after cellular damage. Of the two BAD phospho-serine sites examined in our screen, only the S112 position demonstrated this differential phospho-signaling. Thus, there may be unique requirements/signaling events between the differing RVFV strains.

Of the commonly altered signaling pathways after RVFV infection, the high activation of R-Smads was of interest due to their transcriptional regulation of genes involved in embryonic development as well as homeostasis and plasticity in mature tissues. These evolutionarily conserved factors are activated via the TGF-β ligand superfamily. There are several different members, encoding TGF-β isoforms, BMPs, growth differentiation factors (GDFs), Activins, Nodal, and anti-Müllerian hormone (AMH) that are important for autocrine and paracrine signaling [17, 57–59]. These proteins (*e*.*g*. TGF-β, BMP) can be synthesized as precursors that are then processed during secretion. In many cases, with the most well-studied being TGF-β, these ligands are still in their latent form and requires further processing for the active mature peptide to then bind to its receptor complex. It should also be noted that increased expression of these precursors does not always correlate with increased receptor activity [60]. Thus, there are numerous levels of control in place for bioavailability of these cytokines.

From our study, R-Smad phosphorylation requires active RVFV replication and is impaired in the absence of NSs. Phosphorylation of a number of host proteins, including ATM, p53, Chk2, H2A.X and STAT3, is dependent on NSs [61–63]. However, NSs expression alone was not sufficient to induce R-Smad phosphorylation, indicating that another viral component, presumably present at high levels during viral replication, is necessary to activate Smad signaling. Transfection of cells with poly I:C suppresses TGF-β signaling [64], suggesting that RVFV genomic RNA is not important for stimulating R-Smad activation. The mechanism driving R-Smad activation in the context of infection is still unknown. Based on a prior report and our own observations mRNA export from the nucleus is blocked during RVFV infection [65, 66], thus an increase in cytokine synthesis would likely not be a factor in Smad activation. The proprotein convertases (PPCs) are a family of serine endopeptidases that cleave these secretory protein precursors. In particular, furin, PC5, PC6 and PACE4 have been shown to cleave TGF-β, GDF11, BMP4, and Nodal, respectively [67, 68]. Although, PPCs are important for glycoprotein maturation in a number of viral infections, in the context of RVFV, there are no known requirements for PPC activity to date [69–75]. After cleavage of the precursor, an N-terminal portion, termed latent associated peptide (LAP), remains non-covalently bound with the C-terminal domain of the TGF-β ligand [76–82]. This complex is secreted in association with the latent TGF-β binding protein (LTBP) forming the large latent TGF-β complex or in some cell types, by itself. The large latent TGF-β complex is anchored locally to the extracellular matrix. Release of the mature TGF-β ligand (C-terminal domain) occurs through either conformational changes mediated by α_v_β_6_-integrin or thrombospondin-1 (TSP-1) association or proteolytic cleavage by plasmin, matrix metalloproteinase (MMP)-2/9 or ADAMTS1. Whether there is increased activity of these factors due to RVFV infection remains to be examined.

Another factor to consider is the influence of NSs on IL1R2 mRNA expression. NSs induces the proteasomal degradation of TFIIH subunit p62 through interaction with FBX03, resulting in suppression of host transcription [41, 45, 83–85]. A recent transcriptomic analysis by our group has shown that many genes are upregulated in RVFV infected cells [66], which is in contrast to the typical dogma of NSs shutting down host transcription. However, a confounding factor in these analysis is the presence of uninfected cells and infected cells expressing low levels of NSs. In our work, approximately 25% of HSAECs were uninfected (or had undetectable NP levels) at 9 hpi. Moreover, it is possible that the pool of TFIIH p62 is not completely depleted and/or that another factor is able to compensate for its loss. Future studies could directly address this issue by performing single cell analysis of NSs and IL1R2 expression.

Knockdown of Smads did not impact viral replication (Fig 4). However the importance of Smad activation may be their contribution to pathogenesis. Although mRNA export is blocked in RVFV-infected cells [65], Smad transcription factors may be activated in surrounding uninfected cells through paracrine signaling and/or in cells expressing low levels of NSs. From our promoter analysis, we observed a large number of potential Smad-regulated genes that mediate receptor activation, axonal guidance, and signaling cascades (Table 2). Only one of our ChIP targets, IL1R2, confirmed enrichment of Smad4-containing complexes on their promoter after TGF-β activation or MP12 infection. Furthermore, TGF-β treatment alone only modestly, if at all, increased Smad4 localization on these promoters, *e*.*g*. Col6A3. These results may be indicative of the requirement of BMP or another TGF-β family member for their activation. Also strong Smad complex binding is dependent on association with other transcription factors and coactivator/corepressor complexes. Thus, depending on cell type these additional factors may not be available for Smad-dependent transcriptional activation/repression.

One report where human cases of RVFV infection were divided into non-fatal and fatal clinical outcome demonstrated an elevation in immunosuppressive response in fatal cases [86]. This was indicated by an increase in interleukin 10 and interleukin 1 receptor antagonist (IL1RA). IL1RA binds to the interleukin 1 receptor type 1 (IL1R1) and blocks its activation. Similarly, the IL1R2 acts as a decoy receptor for IL-1 ligands and helps to decrease IL-1 receptor activation (reviewed in [87]). Thus, disruption of IL-1 receptor activation and in turn the inflammatory response may be occurring in RVFV infection.

To date, modulation of Smad signaling in the context of viral infections are almost exclusively in the context of chronic infections, in particular virally-induced cancers [88–91]. Whether this alteration in chronic infections is a consequence of oncogenesis or virally mediated is not known. R-Smad activation is observed following hepatitis C virus (HCV) and hepatitis B virus (HBV) infections, which is not unexpected given that TGF-β is a critical regulator of liver disease [92]. Reports suggests that TGF-β suppresses HCV replication [93] and that inhibitory Smad6 and -7 promote HCV entry [94, 95]. Conversely, TGF-β expression levels and Smad 2/3 phosphorylation were increased in patients with chronic HBV hepatitis and Smad7 mRNA expression was increased in patients who had decreased inflammation due to successful antiviral treatment [96], suggesting that TGF-β signaling is correlated with HBV induced disease. There are only a few examples of Smad 2/3 signaling being activated in acute viral infections, including respiratory syncytial virus (RSV) and human rhinovirus. In RSV infection, Smad2/3 signaling was induced by autophagy activation and loss of Smad2/3 or inhibition of autophagy resulted in reduced interferon-β production [97]. Conversely, human rhinovirus infection results in upregulation of growth differentiation factor 15 (GDF15), which is dependent on IRF-7 and Smad1, indicating a cooperation between Smad and interferon signaling pathways [98]. As such, there is much to be learned about the importance of the Smad signaling for acute viral infections and our study is one of the few examples of R-Smad activation being directly linked to an acute viral infection. Further analysis of how this pathway contributes to potential RVFV pathologies observed in ruminants and in humans is ongoing.

## Disclaimer

Opinions, interpretations, conclusions, and recommendations are those of the author and are not necessarily endorsed by the U.S. Army.

## Supporting information

S1 Fig Flow cytometry gating strategy. One set of representative histograms with gating for RVFV NP staining as depicted by bar graph for Fig 1C.
(TIF)

S2 Fig Smad transcripts decrease over time after RVFV infection. HSAECs (A) and Huh-7 (B) were infected with MP12 (blue) or ZH548 (red) RVFV at an MOI 5. cDNA from RNA lysates was generated and levels of Smad1-7 were analyzed by qPCR. Bars represent means and standard deviations of three to four replicates.
(TIF)

S1 Table Possible Smad-dependent transcripts. RNASeq fold change values for potential Smad-dependent transcripts. The transcripts listed in this table had a Smad matrix similarity of ≥ 0.995.
(XLSX)
