# Correction: Phosphoproteomic analysis reveals Smad protein family activation following Rift Valley fever virus infection

**DOI:** 10.1371/journal.pone.0194633

**Published:** 2018-03-15

**Authors:** Cynthia de la Fuente, Chelsea Pinkham, Deemah Dabbagh, Brett Beitzel, Aura Garrison, Gustavo Palacios, Kimberley Alex Hodge, Emanuel F. Petricoin, Connie Schmaljohn, Catherine E. Campbell, Aarthi Narayanan, Kylene Kehn-Hall

Figs 4, 5 and 6 are incorrect. Errors appear in Fig 4. The image that appears as Fig 5 should be Fig 6, and the image that appears as Fig 6 is a duplicate of Fig 7. The authors have provided corrected versions of Figs 4, 5 and 6 here.

**Fig 4:**
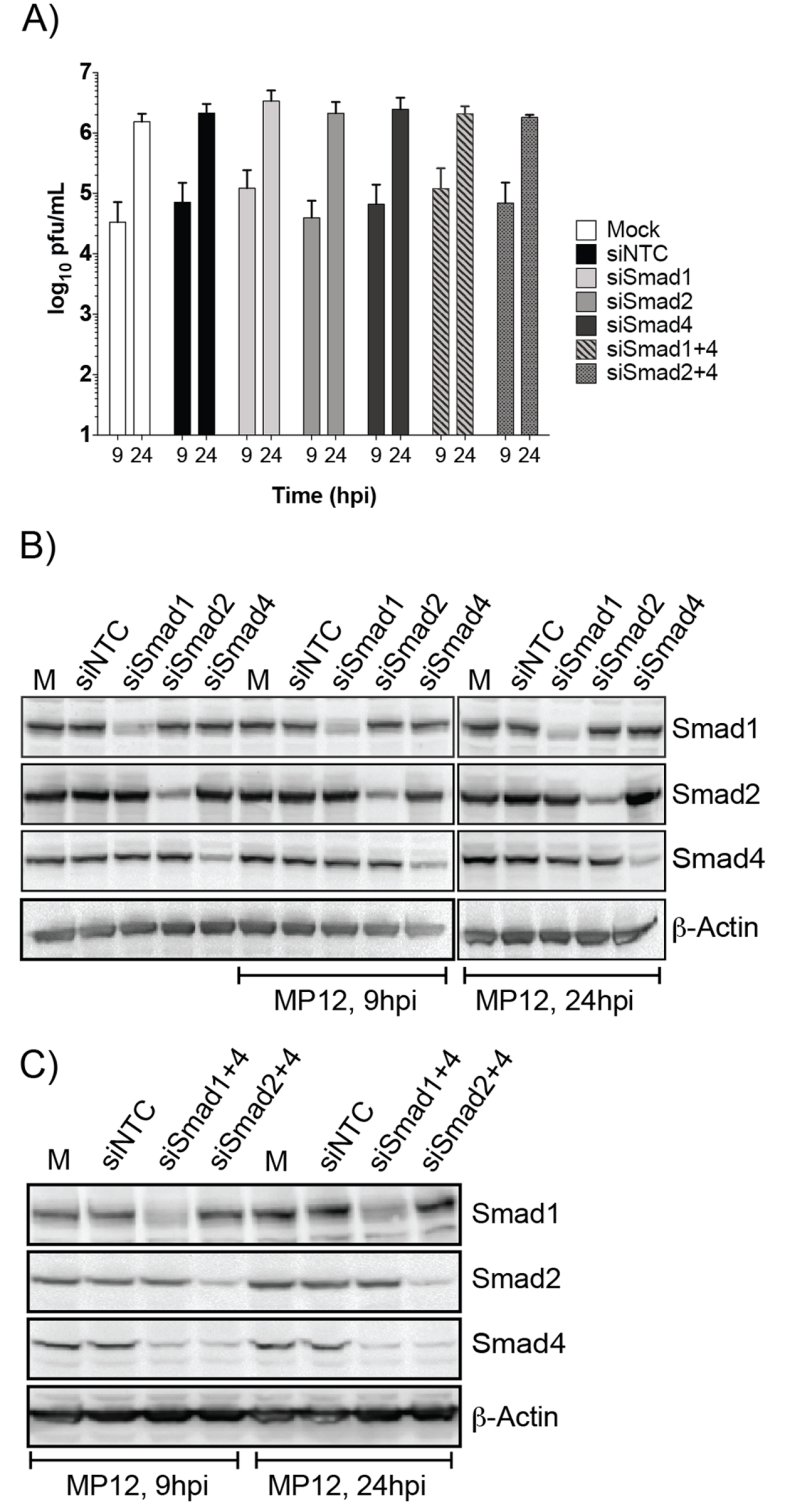
siRNA knockdown of Smad1, -2 and 4 does not impact RVFV replication. HSAECs transfected with media alone (Mock) or siRNAs (75nM) directed against a nontargeting control (NTC), Smad1, -2, or -4 were infected with MP12 virus (MOI 0.1). Both extracellular media supernatants (A) and protein lysates (B,C) were harvested at 9 and 24hpi. A) Infectious viral titers were determined by plaque assay. Data plotted as a bar graph of the mean with standard deviation of four replicates. Protein lysates from single (B) and double knockdowns (C) were analyzed by western blot for actin, total Smad1, -2, and -4 expression.

**Fig 5:**
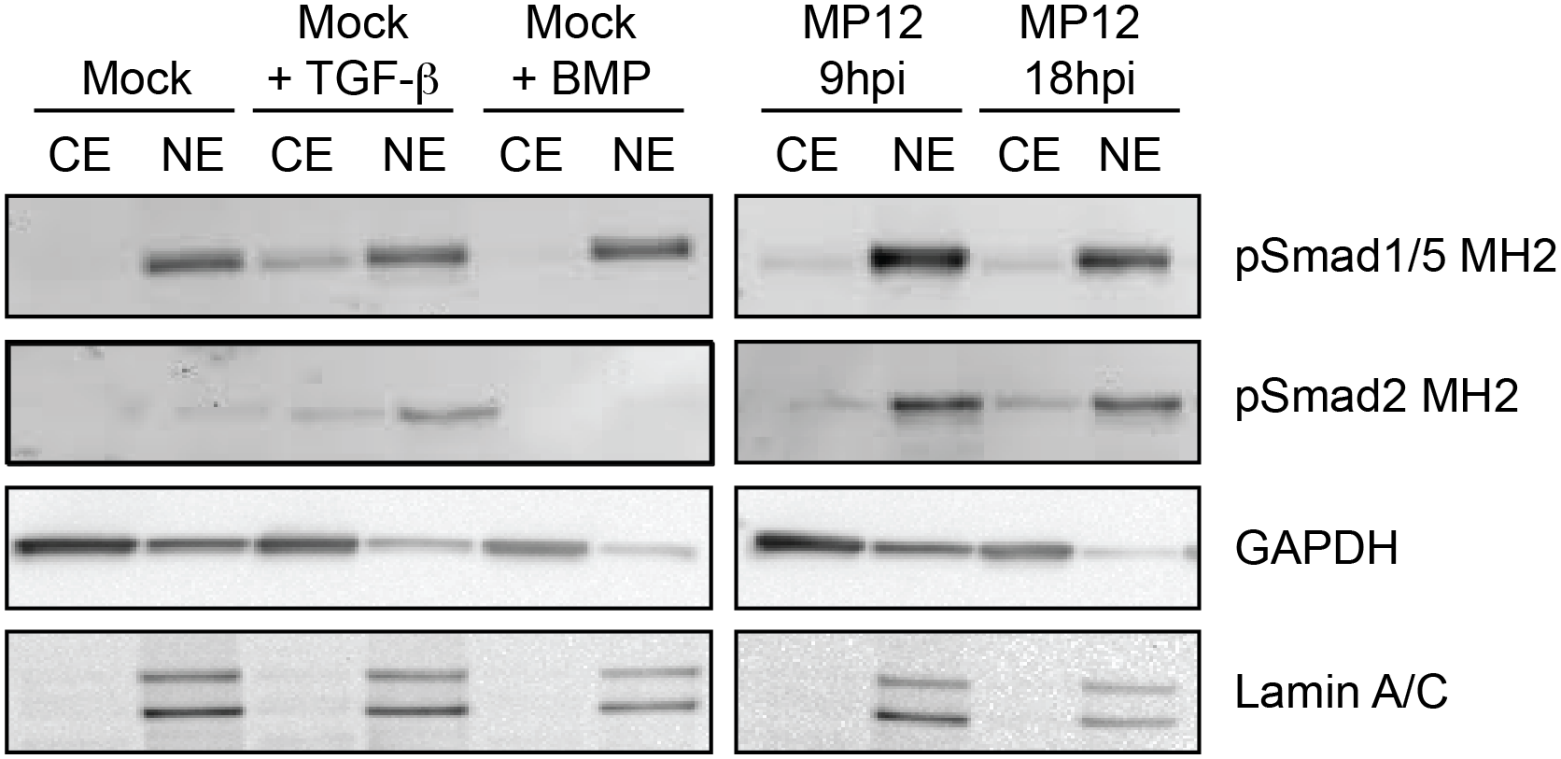
Nuclear localization of phosphorylated Smad proteins. HSAEC cells either alone (Mock), treated with TGF-β3 (50ng/mL, 2hrs), BMP-4 (50ng/mL, 2hrs), or infected with MP12 (MOI 5) for 9 or 18hpi were cell fractionated. 10ug of nuclear extracts (NE) or cytoplasmic extracts (CE) were probed for pSmad1/5 MH2, pSmad 2 MH2, GAPDH and Lamin A/C levels.

**Fig 6:**
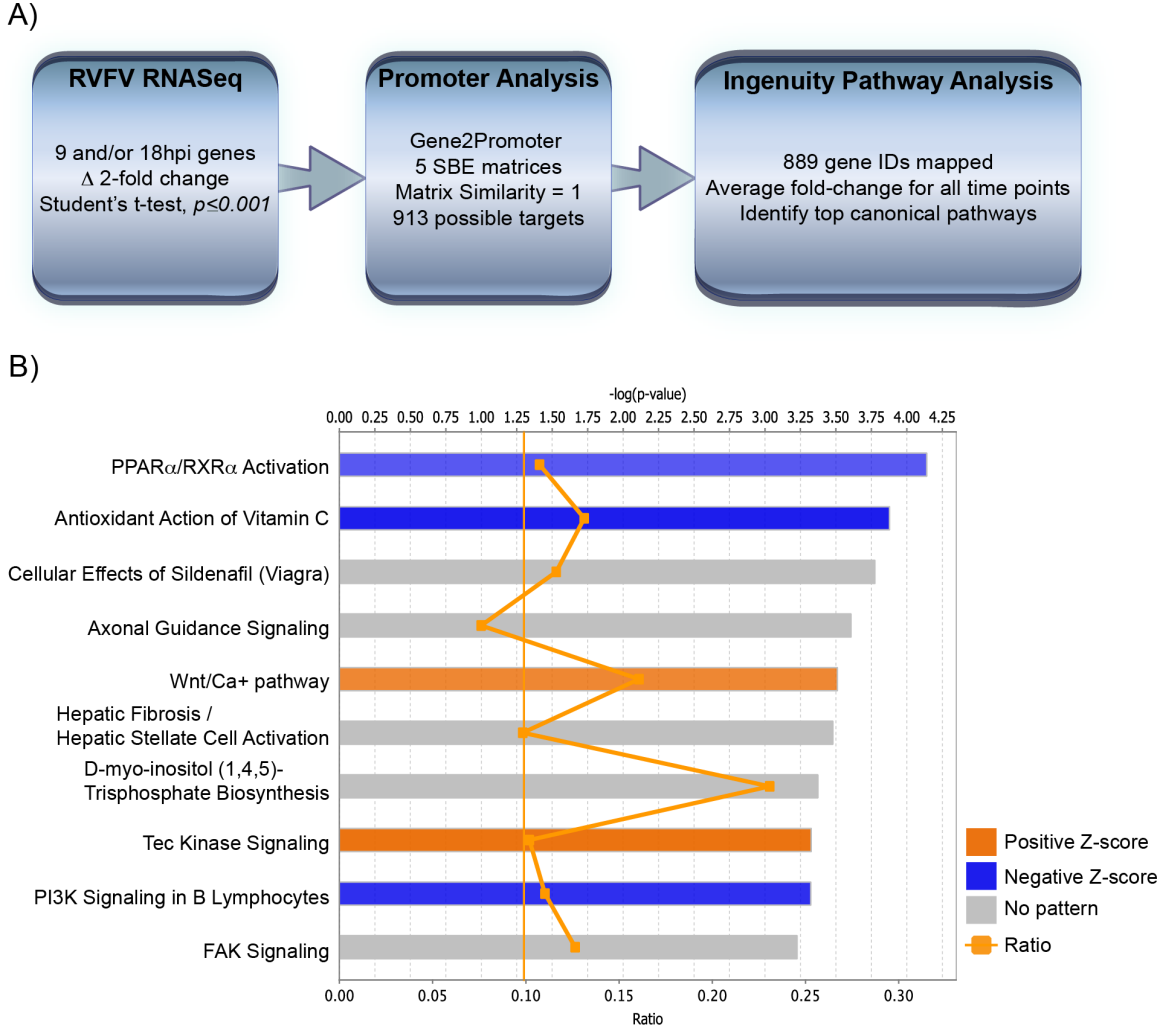
Promoter analysis of RVFV RNASeq yields potential Smad-dependent promoters. A) Schematic depicting data-mining of RVFV RNASeq dataset for Smad-dependent promoters. B) Top ten significantly altered canonical pathways from IPA are shown for the virulent RVFV strain at 18hpi. The top axis indicates the statistical significance calculated using the right-tailed Fisher exact test. Threshold line represents significance cut-off at p = 0.05. Bars represent the level of significance, with orange and blue bars indicating whether the pathway is predicted to be activated or inhibited, respectively (z-score). Pathways where no prediction can be made are colored gray. The ratio (bottom axis) represents the number of molecules identified in a given pathway divided by total number of molecules that constitute that pathway.
