# Supplementary figures and images for: Phosphoproteomic analysis reveals Smad protein family activation following Rift Valley fever virus infection
Source: PLoS One. 2018 Feb 6;13(2):e0191983. doi: 10.1371/journal.pone.0191983 (PMC5800665; doi:10.1371/journal.pone.0191983)

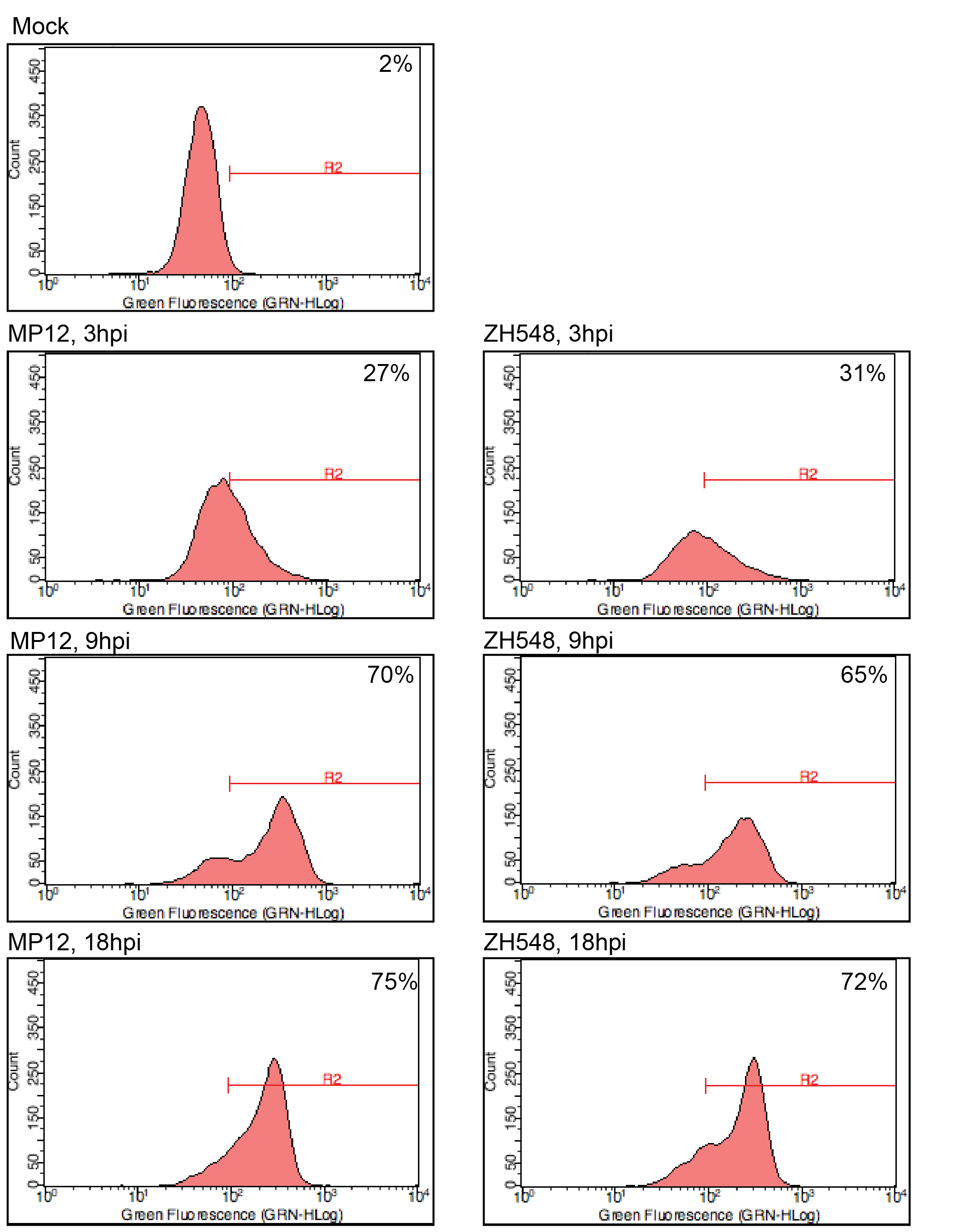

Supplement: S1 Fig — One set of representative histograms with gating for RVFV NP staining as depicted by bar graph for Fig 1C. (TIF) [file pone.0191983.s001.tif]

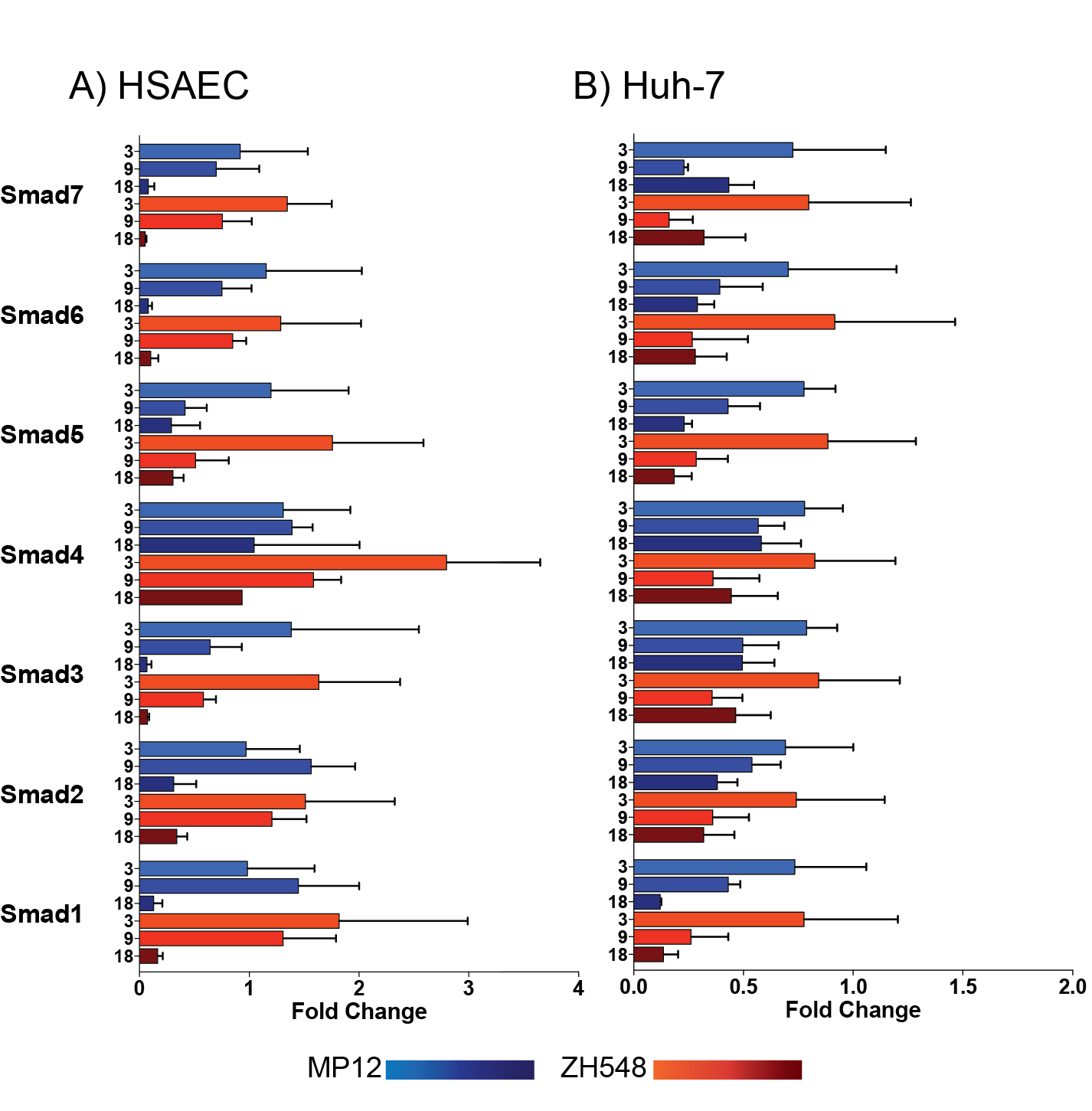

Supplement: S2 Fig — HSAECs (A) and Huh-7 (B) were infected with MP12 (blue) or ZH548 (red) RVFV at an MOI 5. cDNA from RNA lysates was generated and levels of Smad1-7 were analyzed by qPCR. Bars represent means and standard deviations of three to four replicates. (TIF) [file pone.0191983.s002.tif]
